# A Genomic Catalog of Migratory Microbiomes from Wild Birds across China's Habitats

**DOI:** 10.1002/advs.74581

**Published:** 2026-02-26

**Authors:** Yanan Wang, Heqi Wu, Mengqi Qu, Chunge Zhang, Ziqian Xu, Yuhang Pei, Chaochao Zhao, Jiangtao Wang, Sufang Ma, Na Lyu, Xuebin Xu, Yuhai Bi, Baoli Zhu, George F. Gao

**Affiliations:** ^1^ International Joint Research Center of National Animal Immunology College of Veterinary Medicine Henan Agricultural University Zhengzhou China; ^2^ CAS Key Laboratory of Pathogen Microbiology and Immunology Institute of Microbiology Chinese Academy of Sciences (CAS) Beijing China; ^3^ Longhu Laboratory of Advanced Immunology Zhengzhou China; ^4^ Beijing Key Laboratory of Antimicrobial‐Resistant Pathogen Microbiology and AI‐Empowered Containment Institute of Microbiology Chinese Academy of Sciences (CAS) Beijing China; ^5^ State Key Laboratory of Genetic Evolution & Animal Models Kunming Institute of Zoology Chinese Academy of Sciences Kunming China; ^6^ University of Chinese Academy of Sciences Beijing China; ^7^ National Institute for Viral Disease Control and Prevention Chinese Center for Disease Control and Prevention Beijing China; ^8^ Division of Pathogen Testing and Analysis Shanghai Municipal Center for Disease Control and Prevention Shanghai China; ^9^ Center for Influenza Research and Early‐Warning CAS‐TWAS Center of Excellence for Emerging Infectious Diseases Chinese Academy of Sciences Beijing China; ^10^ Chinese Center for Disease Control and Prevention Beijing China

**Keywords:** antimicrobial resistance, antibiotic resistance gene, metagenome‑assembled genome, microbiome, migratory bird, plasmidome, virome

## Abstract

Migratory birds play an important role in the spread of antimicrobial resistance (AMR); however, gaps in surveillance data from vital regions along migratory flyways across China limit the detection of emergent threats. Here, we assembled 340 metagenomes from 52 bird species covering 11 provincial administrative districts in China, presenting a specialized migratory microbial genome and gene catalog to archive the genomic and functional diversity of gut microbiomes in wild birds. This comprehensive migratory bird microbial genome and gene (MBGG) catalog includes 5823 metagenome‐assembled genomes (MAGs), 13 072 plasmid sequences, and 44 974 viral genomes, which represent 1709 candidate species spanning 36 phyla. The catalog also contains over 20 million non‐redundant protein‐encoding genes, the use of which is confirmed by the mining of 15 678 secondary metabolite biosynthetic gene clusters, 1814 known antibiotic resistance genes, and 7219 virulence factors. The number of clinically critical ARGs identified in *Grus japonensis* was the highest, followed by *Cygnus cygnus* and *Sibirionetta formosa*, which indicated that these species are hotspot species of clinically critical AMR dissemination. Moreover, we mapped the profile of bacterial zoonotic/opportunistic pathogens carried by wild birds and evaluated their associations with publicly available genomes. Finally, the precise migratory movements for 10 bird species using a global positioning system tracking system help to assess the movement of microorganisms and AMR risk. Collectively, this valuable resource provides the basis for the integration and unification of global wild bird microbiomes, timely sharing, and assessing the uncertainty of migratory microbiomes in the future.

## 1 Introduction

Wild birds are important reservoirs of pathogenic zoonotic viruses, antimicrobial‐resistant bacteria (ARB), and antibiotic resistance genes (ARGs), posing an extremely serious threat to human and animal health [1, 2, 3, 4, 5, 6, 7]. A recent study analyzed 1834 studies and identified 760 pathogen species in 1438 wild bird species across 38 bird orders [6]. There are nine migratory flyways for birds around the world, which connect different continents and ecosystems. Tens of thousands of migratory birds fly along these routes or gather, especially in the Arctic region. However, a large number of microorganisms, with the help of migratory birds, freely shuttle around the world, becoming migratory microbiomes and bringing potential ecological risks. In particular, pathogens and opportunistic pathogens carried by wild birds are defined here as migratory pathogen repositories. It was estimated that approximately 5.2 billion individual birds live on the planet, covering 9700 bird species (∼92% of all known bird species), which is about six times the number of people on Earth [8]. Thus, the migratory microbiome also constitutes a valuable imprint and resource of the diversity of the microbiome on our planet.

Genome‐resolved metagenomics is a powerful method that enables the recovery of abundant genomes from complex environmental microbial communities [9]. With advances in novel sequencing technologies and bioinformatics tools, genome‐resolved metagenomics has now been applied to more habitat samples and at larger scales, including human microbiome [10, 11, 12, 13], ocean [14], glacier [15], chicken [16, 17], goat [18], pig [19], ruminants [20], horse [21], wild animals [22], pandas [23], soil [24], and environmental samples [25, 26, 27]. These studies have deepened our understanding of the evolutionary trajectories and functions of uncultivated bacteria and archaea, such as antimicrobial peptides, metabolism, and biosynthetic gene clusters (BGCs) [14, 25]. However, the vast number of microorganisms carried by wild birds cannot be readily culturable to date and are identified only through non‐cultured molecular methods. Thus, a metagenomic catalog of migrating microbiomes offers the possibility of large‐scale comparison of genomics and mining novel functional genes. Although sequencing efforts have been undertaken nationally and globally to investigate zoonotic pathogens and antimicrobial resistance (AMR) profiles carried by wild birds, only limited studies have carried out a comprehensive method to evaluate the functional and biosynthetic potential diversity of the migratory microbiomes [28, 29].

The increasing number of infections caused by ARB worldwide is a serious challenge, causing approximately 5 million deaths each year [30]. As we know, diversified multidrug‐resistant (MDR) and extensively drug‐resistant (XDR) bacteria have been listed in the 2024 World Health Organization Bacterial Priority Pathogens List [31]. Recent studies suggest that multiple acquired ARGs confer resistance to last‐line antibiotics have been detected in wild birds [2, 3]. With more than 1400 bird species recorded in China, our understanding of the whole ARGs (resistome) carried by the migratory microbiomes of wild birds is still limited. However, the emergence of bioinformatics tools (such as artificial intelligence, machine learning, and big data) and comprehensive resistance gene databases [32, 33, 34, 35] has enhanced the possibility of mapping a more complete view of ARB and ARG profiles (including known and novel candidate ARGs) carried by wild birds.

**FIGURE 1:**
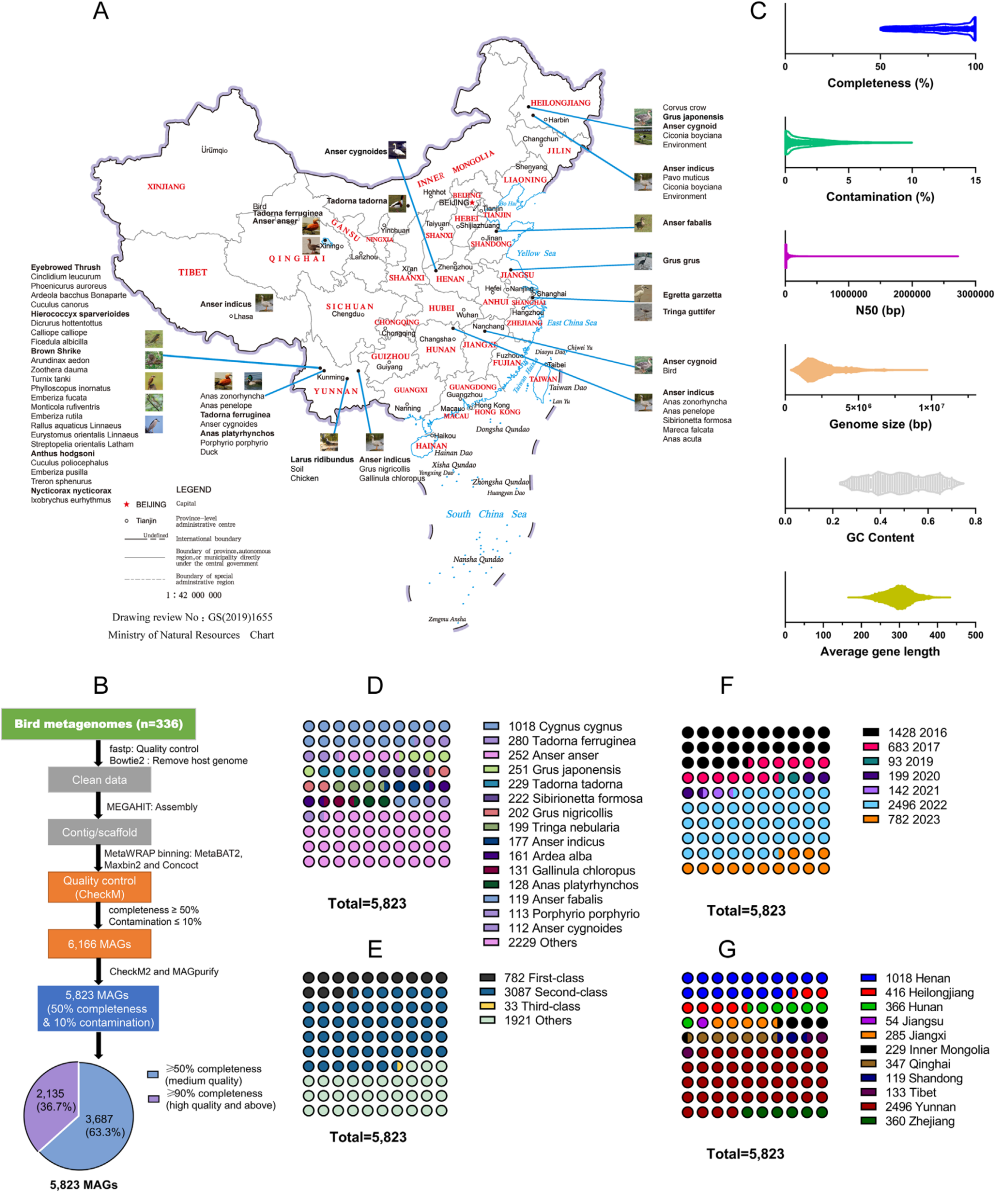
Geographical distribution of migratory metagenome‐assembled genomes. (A) Geographic distribution of metagenomes within each species/origin. (B) A total of 5823 MAGs were recovered from 340 sequenced metagenomes. All retained MAGs were ≥ 50% complete & ≤ 10% contaminated. (C) Distribution of quality metrics across these MAGs (*n* = 5823), showing the first quartile, the minimum, the third quartile, the median value, and the maximum value. (D) Distribution of MAGs across bird species, based on the metadata. The number of MAGs associated with each species (top 15) is indicated next to the plot. (E) Distribution of MAGs across different protection classes of nationally protected animals. (F) Distribution of MAGs across sampling periods. (G) Distribution of MAGs across different sampling locations.

Despite the fact that four of the nine global migration routes cross into China, currently available metagenomic datasets on wild birds are very limited. To address the knowledge gap and the lack of a reference genome catalog on the limited publicly available metagenomes of wild birds across China's habitats, which represent a limited number of species diversity and geographical locations. Here, we performed large‐scale genome‐resolved metagenomics on 340 fresh bird fecal samples and associated samples collected from remote, uninhabited regions across 11 different province administrative districts (Yunnan, Zhejiang, Tibet, Inner Mongolia, Hunan, Qinghai, Henan, Shandong, Jiangxi, Jiangsu, and Heilongjiang) in China to reconstruct 5823 medium‐ and high‐quality metagenome‐assembled genomes (MAGs) covering 52 bird species from China, including national first, second, and others (Figure 1A). These birds covered three major migration routes, east Asian‐Australasian, central Asian, and west Asian‐East African flyway, which are critical for biodiversity and ecosystem health around the world. We present a migratory bird bacterial genome and gene (MBGG) catalog, which contains data on the species, genome, and gene levels. To obtain an overview of the virome and plasmidome in the migratory microbiome from wild birds, we analyzed these microbiome data using open‐source workflows, integrated tools for reconstruction of plasmid and viral sequences, taxonomic classification, and functional annotation. We also identified 44 193 species‐level viral operational taxonomic units (vOTUs) from these metagenomes as part of the genomes in the MBGG catalog. The MBGG catalog represents 1709 novel candidate bacterial and archaeal species‐level operational taxonomic units, as well as over 20 million non‐redundant genes. To demonstrate and mine the value of these genomic resources, we used the MBGG catalog and metagenomic read mapping analysis to explore the bird microbial diversity, biosynthetic capacity, AMR profiles, and mobile genetic elements (MGEs), as well as the evaluation of potential public health risks of migratory pathogenic microorganisms. We also employed the Global Positioning System (GPS) tracking system to monitor 10 species of wild birds and obtain migration routes, which helps us understand the migration of microorganisms and AMR genes carried by wild birds. In addition, we provide a methodological blueprint under the One Health strategy for exploring the diversity and transmission routes of migratory zoonotic/opportunistic pathogens carried by wild birds, for assessing the uncertainty of migratory microbiomes.

## 2 Results

### 2.1 Construction of a Migratory MAG Catalog of Wild Birds Across China's Habitats

We performed metagenomic assembly and binning on 340 metagenomes (Table S1) from diverse wild bird species (*n* = 316) and associated poultry samples (*n* = 19) in natural reserves, as well as habitat soil environments (*n* = 5) located in 11 different provincial administrative districts in China (Figure 1A), to recover 5823 MAGs (Figure 1B). All these migratory MAGs meet the minimum medium‐quality standard of MAG criteria (completion ≥50%, contamination ≤10%) with an average completeness of 81.92% and a mean contamination rate of 1.99% (Figure 1C). The genome sizes of these migratory MAGs ranged from 0.38 to 9.25 Mb, and GC% varied from 23% to 75% (Figure 1C; Table S2). Therefore, we referred to these 5823 MAGs as the migratory MAG catalog of birds across China's habitats. This migratory catalog of MAGs contains representatives from 52 different species belonging to 47 genera, 22 families, and 10 orders, within the Aves class (Figure 1D) and involving National first‐class, second‐class, and other species (Figure 1E). To our knowledge, the migratory MAG catalog MBGG we built is the largest, representative dataset in China to date and globally, and the samples span an eight‐year period, from 2016 to 2023 (Figure 1F), covering 11 provincial administrative regions in China (Figure 1G).

### 2.2 The MBGG Catalog Expands Our Insight into Previously Unidentified Bacterial and Archaeal Species

**FIGURE 2:**
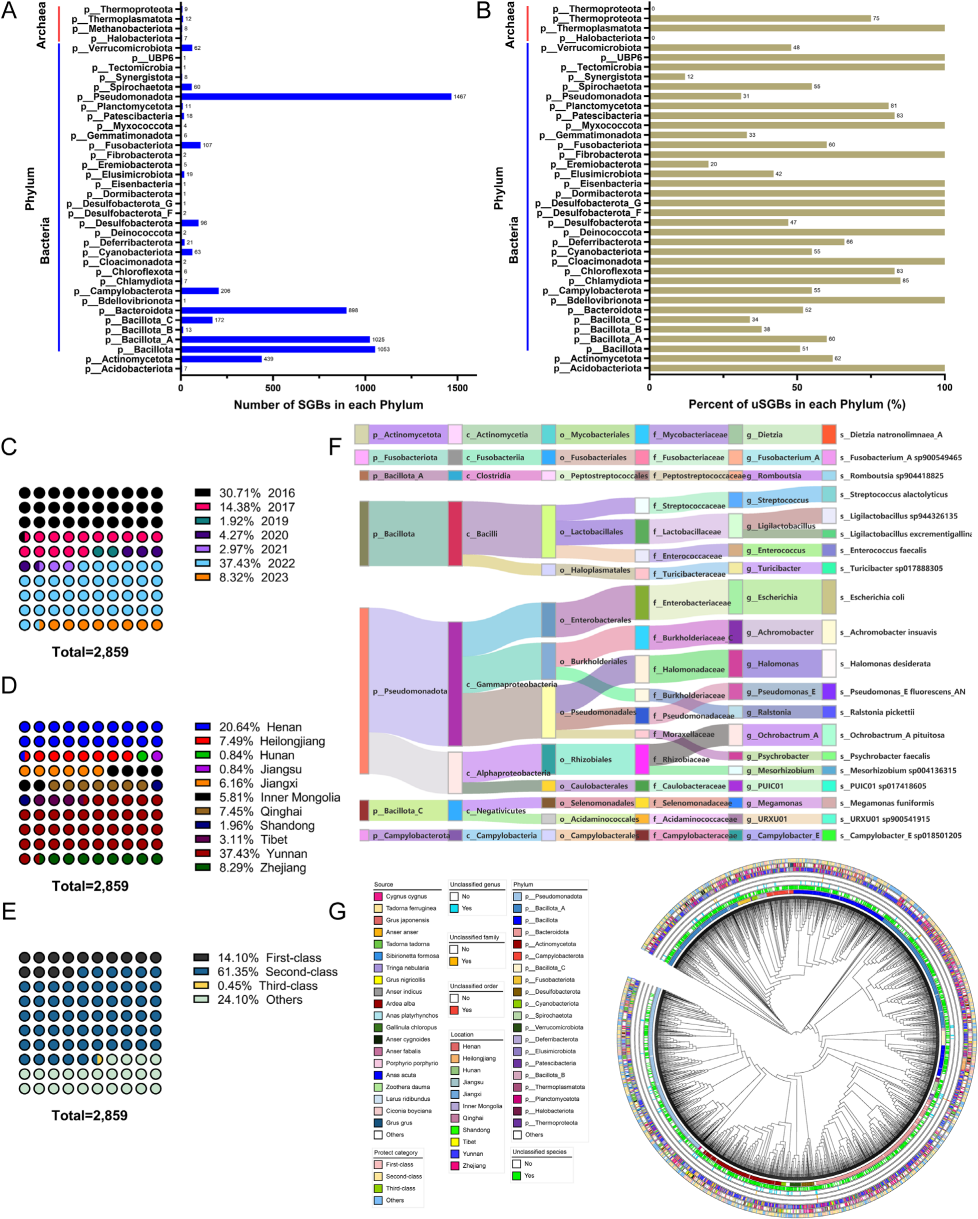
The migratory MAGs substantially expand the diversity of bird gut microbes. (A) Number of MAGs assigned to each phylum. A total of 5823 MAGs were recovered from 340 sequenced metagenomes. All retained MAGs were ≥ 50% complete & ≤ 10% contaminated. (B) Percentage of 2859 uSGBs in each phylum of both bacterial and archaeal clades. (C) Distribution of the fraction of uSGBs in each sample by location. (D) Distribution of uSGBs across different national classes of protected animals. (F) Sankey diagrams showing the distribution of MAGs at different taxonomic levels from species to phylum. A total of 20 dominant species were summarized and shown. (G) The phylogenetic relationship between the 5823 MAGs in the migratory genome catalog and their taxonomic classification according to GTDB–Tk. The annotations from inside to outside represent annotations at the species level (different colors represent different phyla), unclassified species, unclassified genus, unclassified family, location, source, and protection category. Abbreviations: SGBs, species‐level genome bins; kSGB, known SGBs; uSGBs, unknown SGBs; MAGs, metagenome‐assembled genomes; GTDB, Genome Taxonomy Database.

Based on taxonomic classifications from the GTDB‐Tk [36], these migratory MAGs represent 38 phyla, 64 classes, 136 orders, 268 families, 754 genera, and 959 known species (Figure 2A; Table S3). A total of 5787 bacterial and 36 archaeal MAGs were obtained, respectively (Figure S1). The migratory MBGG catalog of MAGs is dominated by Proteobacteria (synonym Pseudomonadota), Bacillota, Bacillota_A, Bacteroidota, Actinomycetota, and Campylobacterota, whereas the number of MAGs belonging to the Bdellovibrionota, Dormibacterota, Eisenbacteria, Tectomicrobia, and Desulfobacterota_G was very small, with only one MAG for each phylum being obtained. A total of 2859 MAGs do not have representative genomes in the GTDB (using the ANI threshold of 95%) at present, therefore representing new candidate lineages. These unreported MAGs belonged to 36 phyla (Figure 2B), which were dominated by three novel orders, 11 novel families, 149 novel genera, and 1709 novel species (unknown species‐level genome bins, uSGBs) (Table S3).

Several MAGs were identified in birds from different sampling periods and locations, indicating that a few species have a broad habitat environment. From 2016 to 2023, covering 11 different provincial administrative districts, all identified at least one novel microbial species (Figure 2C,D). The most novel species have been discovered in Yunnan (37.43% of 2859), followed by Henan (20.64%), Zhejiang (8.29%), Heilongjiang (7.49%), and Qinghai (7.45%) (Figure 2D). All 50 bird species carried at least one new lineage, and the most predominant is *Whooper swans* (*n* = 590), followed by *Tadorna ferruginea* (*n* = 184), *Tadorna tadorna* (*n* = 166), *Anser anser* (*n* = 151), and *Ardea alba* (*n* = 135). These new lineages were mainly distributed in national second‐class protected birds (61.35%), followed by first‐class (14.10%) (Figure 2E). Then, Sankey plots summarized the distribution of 20 dominant species at different taxonomic levels from species to phylum (Figure 2F). In addition, we constructed a phylogeny of the 2987 non‐redundant MAGs (Figure 2G) based on concatenated marker genes. The phylogenetic tree also supported that the MBGG catalog is the most diverse dataset of wild birds published to date worldwide, enlarging the scope of microbial diversity from birds.

### 2.3 The MBGG Catalog is a Powerful Resource for Mining Functional Potential

To capture the function genes of the entire migratory microbiome of 50 bird species, the microbial gene catalog was built using all the assembled contigs. A total of 74 076 453 open reading frames (ORFs) were predicted based on prodigal prediction, which was then de‐replicated into 20 062 410 gene clusters using the criteria of identity 95% and overlap 90%. Based on a 100% identity criterion for removing redundancies, these open reading frames remain at 27 515 963, accounting for 37% of the total. Thus, this non‐redundant gene catalog can be used as a reference dataset for future migratory metagenome mining analysis. We used the eggNOG‐mapper v2.1.13 [37] to annotate the functional potential of the non‐redundant gene catalog and found that only 9 073 208 (45.22%) of the genes were annotated, whereas the remaining genes (54.78%) could not be annotated for functions.

**FIGURE 3:**
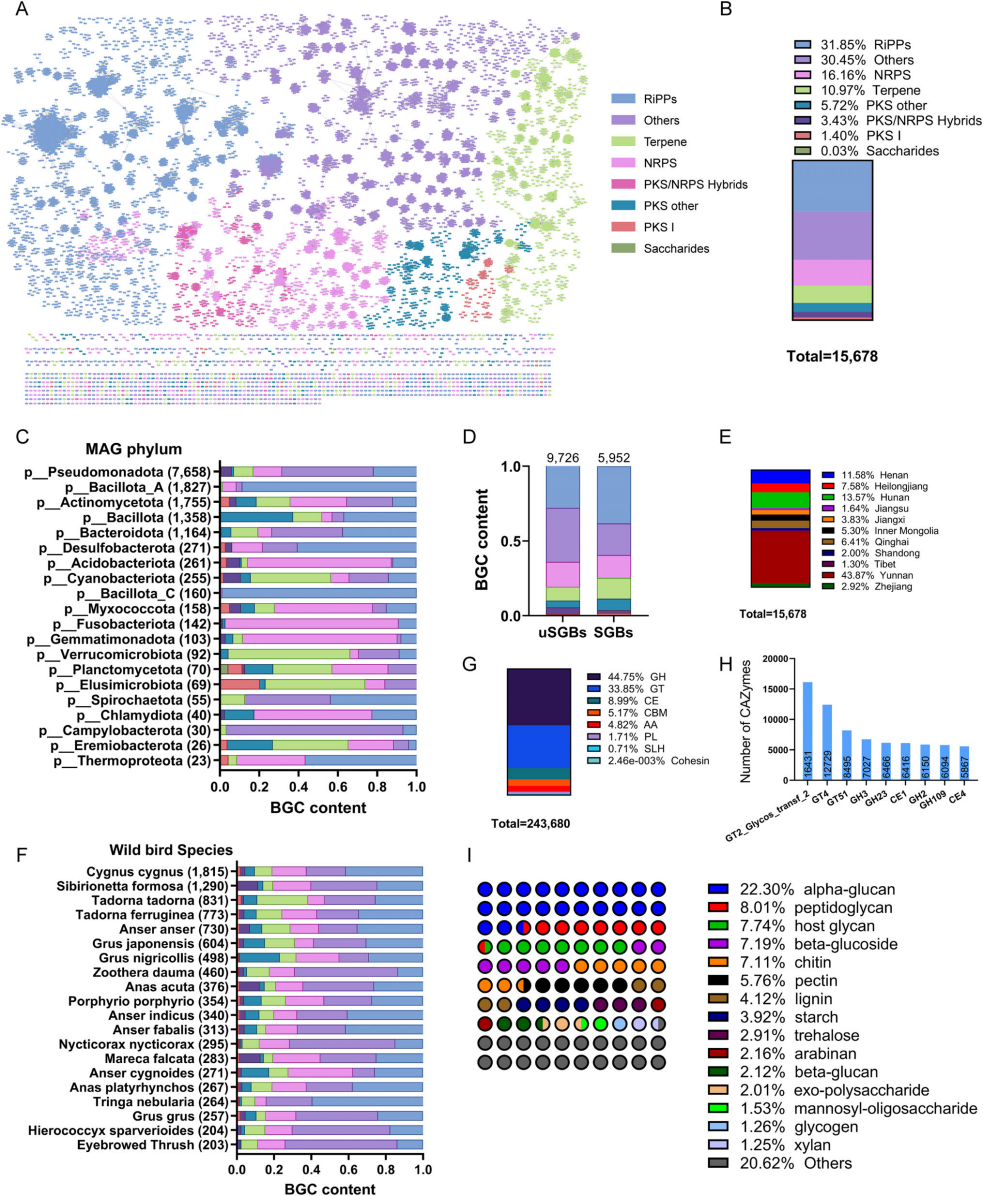
Biosynthetic gene clusters and carbohydrate‐active enzymes recovered from the migratory MAG catalog. (A) A detail of the BiG‐SCAPE network for BGCs sequences. (B) The relative frequency of BGC types across the migratory MAGs. (C) The relative frequency of BGC types across predominant phyla. (D) The relative frequency of BGC types in known and unknown species. (E) The geographical distribution of BGC types recovered from the migratory MAGs. (F) The relative frequency of BGC types across predominant wild bird species (*n* = 20). (G) Relative frequency of carbohydrate‐active enzyme (CAZyme) classes identified in the MBGG catalog. (H) The top 10 most abundant CAZyme subclasses identified in the MBGG catalog. AA: Auxiliary Activities, CBM: Carbohydrate‐Binding Module, CE: Carbohydrate Esterase, GH: Glycoside Hydrolase, GT: Glycosyl Transferase, PL: Polysaccharide Lyase. (I) the substrate type for CAZymes. [Correction added on 28 February 2026 after first online publication: Figure 3A has been updated in this version.]

The MBGG catalog provides a potential opportunity to mine the secondary metabolite biosynthetic gene clusters (BGCs) encoded within this precious migratory microbiome dataset. We identified 15 678 putative BGC regions from the MBGG catalog using AntiSMASH v7.1.0.1 [37] (Table S4). The predicted secondary metabolite BGCs were categorized into eight classes (Figure 3A; Figure S2). Of these, BGCs synthesizing ribosomally processed peptide (RiPP) clusters were the most diverse. A total of 4932 (31.85%) RiPP clusters were identified from 18 phyla, 2522 (16.16%) NRPS from 18 phyla, 1699 (10.97%) Terpene from 16 phyla, 884 (5.72%) polyketide synthase (PKS) other clusters from 15 phyla, 539 (3.43%) PKS/NRPS hybrid clusters from 13 phyla, and 216 (1.40%) PKS I clusters from 13 phyla (Figure 3B).

Proteobacteria were the most likely target for antibiotic mining in the MBGG catalog, with 1677 RiPPs, 1124 NRPS, 39 5 PKS–NRPS hybrid BGCs, and 153 PKS identified (Figure 3C). Bacillota had high numbers of RiPPs (more than 46.14% of their BGCs identified), while Verrucomicrobiota and Elusimicrobiota contained relatively high numbers of terpene clusters (61.96% and 50.72% of their BGCs, respectively). In addition, Fusobacteriota, Gemmatimonadota, and Acidobacteriota had unusually high numbers of NRPS (88.03%, 78.64%, and 73.18% of their BGCs, respectively). Of these BGSs identified, 62.04% and 37.96% originated from uSGBs and SGBs, respectively. The proportions of RiPPs, Terpene, and PKS others were higher in SGBs than in uSGBs, while the proportions of PKS/NRPS Hybrids and others were more abundant in uSGBs than in SGBs (Figure 3D). The three dominant locations for the distribution of secondary metabolite BGCs were Yunnan (43.78%), Hunan (13.57%), and Henan (11.58%) (Figure 3E). The majority of BGCs were identified in national first‐class protected birds, and the dominant was *Cygnus cygnus* (*n* = 1815), followed by *Sibirionetta formosa* (*n* = 1290), *Tadorna tadorna* (*n* = 831), *Tadorna ferruginea* (*n* = 773), and *Anser anser* (*n* = 730) (Figure 3F). Therefore, the MBGG catalog is a potentially important source of novel secondary metabolite BGCs and provides a valuable resource for mining BGC evolution in migratory microbiomes from wild birds.

To further investigate the functional potential of these migratory MAGs, a total of 12 960 273 ORFs in these MAGs were annotated. 51.99% (*n* = 6 738 640) and 1.88% (*n* = 243 680) of these ORFs matched known functions according to the Kyoto Encyclopedia of Genes and Genomes (KEGG) and CAZyme databases, respectively. These results suggested that about half of these genes may code for candidate novel functions. These CAZyme‐encoding ORFs were classified into eight classes (Figure 3G; Table S5). The most abundant class was glycoside hydrolase (GH) (44.75%) among the migratory MAGs, followed by glycosyltransferase (GT) (33.85%), carbohydrate esterase (CE) (7.99%), carbohydrate‐binding module (CBM) (5.17%), and auxiliary activities (AA) (4.82%) (Figure 3G). The three most dominant GTs were GT2_Glycos_transf_2, GT4, and GT 51, accounting for 6.74%, 5.22%, and 3.49%, respectively (Figure 3H). The substrate types of these CAZymes were categorized into 68 types; the top five were alpha‐glucan (22.30%), peptidoglycan (8.01%), host glycan (7.74%), beta‐glucoside (7.19%), and chitin (7.11%) (Figure 3I). These results have expanded our understanding of the differences in carbohydrate metabolism in the gut microbiomes of different bird species.

### 2.4 The MBGG is a Powerful Tool for Identifying the Migration of Clinically Important ARGs

**FIGURE 4:**
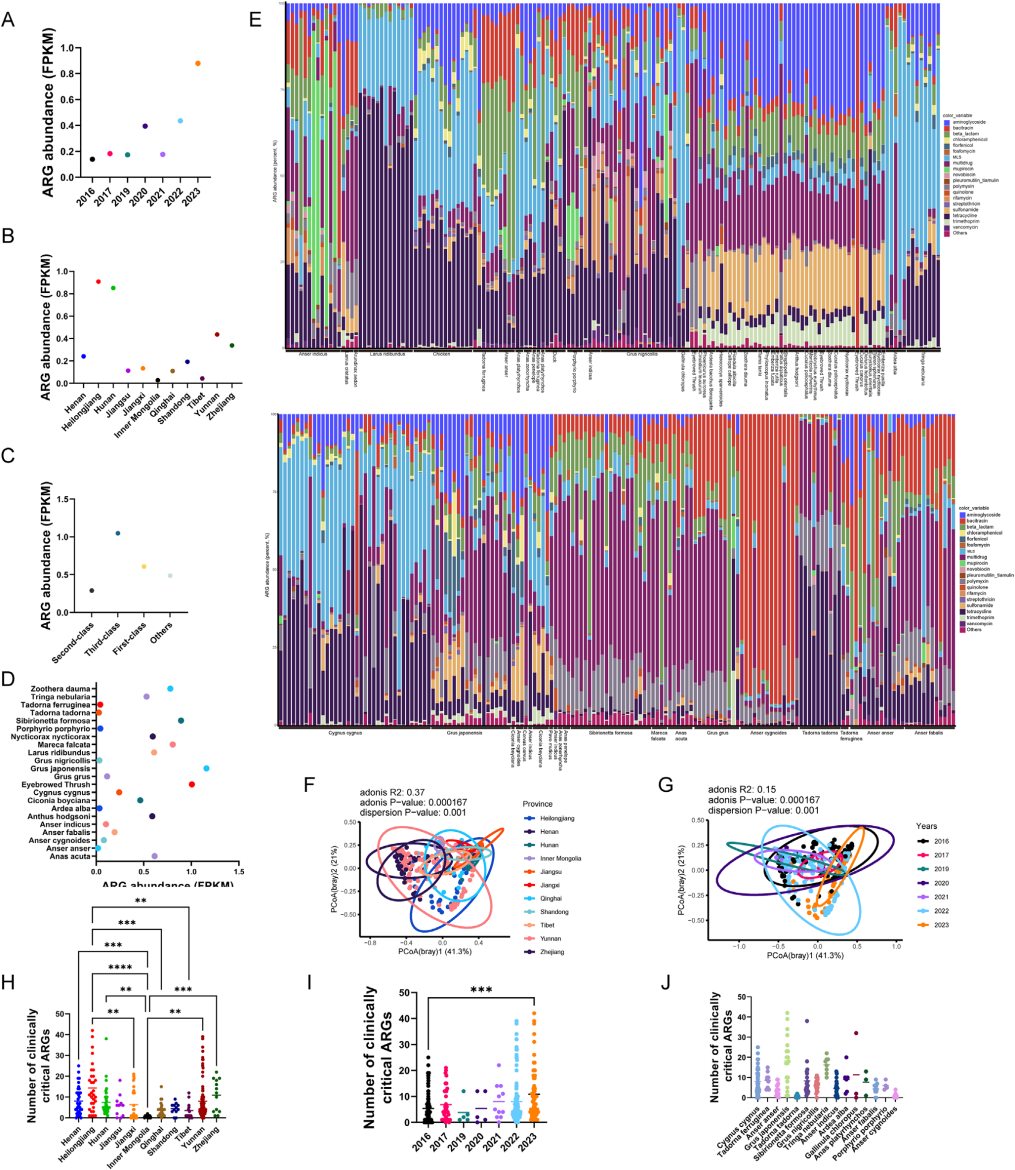
Overview of ARG abundance and composition. (A) Plots showing the average AMR level per sample (the total AMR levels/samples), stratified by sampling periods. From read mapping to the ARG‐OAP3 database, ARG abundance was calculated for each reference gene in each sample. (B) Plots showing the average AMR level per sample, stratified by provinces. (C) Plots showing the average AMR level per sample, stratified by national class protected animal. First‐class: national first‐class protected animal; second‐class: national second‐class protected animal; third‐class: national third‐class protected animal. (D) Plots showing the average AMR level per sample, stratified by host species. (E) Stacked bar chart of ARG abundance per type per sample (host species, x‐axis), proportional to the total ARG abundance within each sample. Note that a total of fifty bird species were summarized. MLS: macrolide‐lincosamide‐streptogramin. (F) Principal coordinate analysis (PCoA) of antibiotic resistance profiles from different geographical locations based on Bray–Curtis distance. (G) PCoA of antibiotic resistance profiles from different sampling periods based on Bray–Curtis distance. (H) Number of clinically critical ARGs each sample identified among different geographical locations. (I) Number of clinically critical ARGs each sample identified among different sampling periods. (J) Number of clinically critical ARGs each sample identified in different species (the top 15).

In this work, we first analyzed the abundance and diversity of known ARGs from 29 major resistance gene classes. The total AMR gene abundance varied significantly across samples, depending on the year of collection, the province of location, and the bird species. In general, the highest AMR levels were found in 2023, followed by 2022, 2020, and others (Figure 4A; Figure S3). Wild bird samples collected in Heilongjiang showed the highest AMR level, followed by Hunan (Figure 4B). AMR levels were higher in birds listed in the national third‐class protected animals than in other classes (Figure 4C). However, as for the top 22 bird species, the highest AMR levels (average of each bird species) were found in *Grus japonensis*, followed by *Eyebrowed Thrush*, *Sibirionetta formosa*, *Larus ridibundus*, and *Zoothera dauma* (Figure 4D).

Then, we summed the ARG abundance to the corresponding antibiotic class level for each to explore major patterns across species; the relative percentages of AMR per drug across 52 species were varied. (Figure 4E; Table S6). In general, tetracycline AMR levels were more abundant in *Larus ridibundus*, *Cygnus cygnus*, *Tadorna tadorna*, *Anser indicus*, *Eurystomus orientalis*, chicken, and duck than in other bird species samples. MLS‐related AMR levels were more abundant in *Ardea alba*, *Tringa nebularia*, *Gallinula chloropus*, *Cygnus cygnus*, and *Larus ridibundus*. Bacitracin AMR levels were more abundant in *Anser cygnoides*, *Eyebrowed Thrush*, and *Grus japonensis*. Beta‐lactam AMR levels were more abundant in *Anser anser*, *Tadorna ferruginea*, *Anser indicus*, *Sibirionetta formosa*, and *Mareca falcata*. Polymyxin AMR levels were more abundant in *Grus grus*, *Anser fabalis*, *Tadorna tadorna*, *Pallus aqaaticus*, *Eyebrowed Thrush*, *Lanius cristatus*, *Arundinax aedon*, *Grus japonensis*, *Sibirionetta formosa*, *Anas zonorhyncha*, *Anas penelope*, *Anas acuta*, and *Anser anser*. Multidrug AMR levels were more abundant in *Sibirionetta formosa*, *Corvus corvus*, *Pallus aqaaticus*, *Anas zonorhyncha*, *Cygnus cygnus*, *Eyebrowed Thrush*, *Anas acuta*, *Mareca falcata*, *Anas penelope*, *Sibirionetta formosa*, *Phoenicurus auroreus*, *Grus grus*, *Anser cygnoides*, *Grus nigricollis*, *Anser fabalis*, and *Grus japonensis*. In different habitats, different wild birds showed similar resistome composition; class‐level abundances can be found in Table S6.

Overall, 1814 known ARG types were identified in migratory microbiomes (Table S7). We also performed principal coordinate analyses for ARGs in different geographical locations (Figure 4F) and sampling periods (Figure 4G). These results indicate that the ARGs carried by the gut microbiomes of wild birds collected in different habitats and at sampling periods (from 2016 to 2023) show clustering differences to some extent. Notably, multiple resistance genes, for example, NDM, IMP, OXA‐48, VIM‐1, MCR, *tet*(X4), conferring resistance to the clinically critical antibiotics and last‐line antibiotics, were identified in a variety of migratory wild birds. Among these 5823 MAGs, a total of 1046 (17.96%) MAGs carried at least one ARG, from 1 to 50 ARGs per genome, for example, *cfrA*, *FosA3*, *optrA*, *mcr‐1*, and *bla*_CTX‐M‐65_. Among these bird species, clinical critical antibiotic resistance markers were more abundant in *Grus japonensis*, followed by *Cygnus cygnus*, *Sibirionetta formosa*, *Anser indicus*, *Anas zonorhyncha*, and *Tadorna ferruginea* (Table S8). For example, carbapenem resistance genes, *bla*_VIM‐1_, *bla*_NDM_, *bla*_IMP_, and *bla*_OXA‐48_ were identified in 46, 2, 4, and 6 bird species, respectively. Tigecycline resistance genes *tet*(X3), *tet*(X4), *tet*(X5), and *tet*(X6) were detected in 20, 9, 15, and 18 bird species, respectively. Mobile colistin resistance gene, *mcr‐1*, was identified in 11 species. Oxazolidinone resistance *optrA*, *cfr(E)*, and *poxtA* were identified in 22, 16, and 14 species, respectively. In addition, the recently discovered sulfonamide resistance gene *sul4* was identified in 14 species.

The number of clinically critical ARGs detected in Heilongjiang was the highest, followed by Zhejiang, Yunnan, Henan, and Hunan (Figure 4H). Higher diversity of clinically critical ARGs was observed in 2023 than in 2016 (Figure 4I). In addition, the number of clinically critical ARGs identified in *Grus japonensis* (*n* = 78) was the highest, followed by *Cygnus cygnus*, *Sibirionetta formosa*, *Anser indicus*, *Anas zonorhyncha*, and *Tringa nebularia* (Figure 4J; Table S8), which indicated that these species are hotspot species of clinically critical AMR dissemination.

### 2.5 Construction of a Plasmid Catalog from Wild Birds

A total of 13 072 contigs were recovered in 604 plasmids using sequence content‐aware plasmid peeler (SCAPP) [38] and verified using other tools for classification and functional gene annotation (for example, PlasClass [39] and MOB‐suite [40]). The genome sizes of these plasmids ranged from 1.49 to 217.21 kb, and GC% varied from 23% to 68% (Figure S4A,B and Table S9). Therefore, we referred to these plasmids as the migratory plasmid catalog of wild birds across China's habitats. Among the ORFs encoded on these plasmids, a variety of ARGs, plasmid mobility genes, DNA replication proteins, called the replication origin (oriC) types, and virulence genes were annotated. We classified plasmid lifestyles into mobilizable (65.07%), conjugative (9.27%), and non‐mobilizable (25.66%) plasmids (Figure S4C) based on the presence or absence of plasmid mobility genes. The dominant replicon type is Col(MG828) (5.63%), followed by rep_cluster_2350 (4.47%) and ColpVC (3.31%) (Figure S4D). Inc11 (1.16%) and IncX1 (1.16%) were the top two dominant Inc‐type plasmids.

We then investigated the association between plasmids and the potential hosts and found that most of these plasmids connected with the Proteobacteria (59.11%), Firmicutes (9.27%), and Bacteroidota (synonym Bacteroidetes) (8.77%) (Figure S4E), indicating that these three phyla may be likely the predominant plasmid hosts in the migratory bird gut microbiomes. We also found that numerous plasmids in migratory microbiomes exhibited similarities to published complete plasmids and draft genomes from *Enterobacteriaceae*, *Acinetobacter*, *Escherichia*, *Aeromonas*, and *Pseudomonas* (Figure S4F). Therefore, these bacteria, especially Proteobacteria, seem to be the primary hosts for the gut plasmidome in migratory birds in this study. For 11 different geographical locations, large variations in the prevalence of plasmid‐associated hosts were observed (Figure S4G). These plasmid‐linked bacteria were mainly identified in samples from Yunnan, Heilongjiang, Hunan, and Henan. The number of plasmids linked to hosts was primarily from *Grus japonensis*, *Grus nigricollis*, *Cygnus cygnus*, *Anas acuta*, *Anser indicus*, and *Sibirionetta formosa* (Figure S4H). Overall, these results indicated that the linkages between plasmids and their hosts varied in the gut microbiomes of different bird species.

### 2.6 Virulence Factors in the MBGG Catalog

A total of 10 588 and 7219 potential virulence factors (VFs) (Tables S10 and S11) were identified based on Virulence Factor Database (VFDB) [41] and the expanded Virulence Factor database (VFDB 2.0) [42]. These VFs were mainly associated with housekeeping (35.64%), secretory (25.74%), colonization (20.49%), and immune evasion (9.39%) by abundance (Figure S5A). VFs were identified in 810 migratory MAGs, with each genome harboring between one and 216 VFs. These VFs were predominantly from Proteobacteria (*n* = 5781), Campylobacterota (*n* = 864), and Bacillota (*n* = 510) (Figure S5B). The number of VFs was higher in *Sibirionetta formosa*, followed by *Grus japonensis* (*n* = 947), *Larus ridibundus* (*n* = 560), *Mareca falcata* (*n* = 457), and *Cygnus cygnus* (*n* = 404). Of these, 47.83%, 9.21%, and 10.08% were species‐specific, plasmid‐borne, and prophage‐borne VFs (Figure S5C–E). The majority (97.70%) of VFs identified in SGBs could be classified into known species according to GTDB, while the remaining set (2.30%) was distributed in uSGBs (Figure S6).

### 2.7 The MBGG Catalog Helps to Understand the Dissemination of Zoonotic or Opportunistic Pathogens

Here, we mapped the composition and relative abundance profiles of microbial communities in 50 bird species (Table S12) using the MetaPhlAn4 with the latest database [43]. Generally, the total abundances of microbial species across samples, depending on birds in different sampling periods and geographical locations, did not show significant clustering differences (Figure S7A,B). The microbial communities in different bird species showed different clustering to some extent (Figure S7C). Multiple zoonotic or opportunistic pathogens were identified in these bird metagenomes (Table S13). For example, the zoonotic pathogen, *Campylobacter jejuni*, was observed in 61 samples distributed across 17 species. *Chlamydia psittaci* was identified in two species, including *Cygnus cygnus* and *Ardeola bacchus Bonaparte*. *Salmonella enterica* was observed in 5 bird species, including *Grus grus*, *Anthus hodgsoni*, *Grus japonensis*, *Phoenicurus auroreus*, and *Anser indicus*. *Yersinia enterocolitica* was observed in 8 samples originating from 3 species, including *Anser indicus*, *Anser fabalis*, and *Gallinula chloropus*. The opportunistic pathogen, *Clostridium perfringens*, was observed in 60 samples distributed across 22 species. *Morganella morganii* was detected in 12 samples across 5 bird species, including *Grus japonensis*, *Tadorna tadorna*, *Ciconia boyciana*, *Corvus corvus*, and *Eyebrowed Thrush*. In addition, *Cryptococcus neoformans* has been identified in *Larus ridibundus* fecal samples. These results have significantly enlarged our understanding of zoonotic or opportunistic pathogens carried by migratory birds across China's habitats.

**FIGURE 5:**
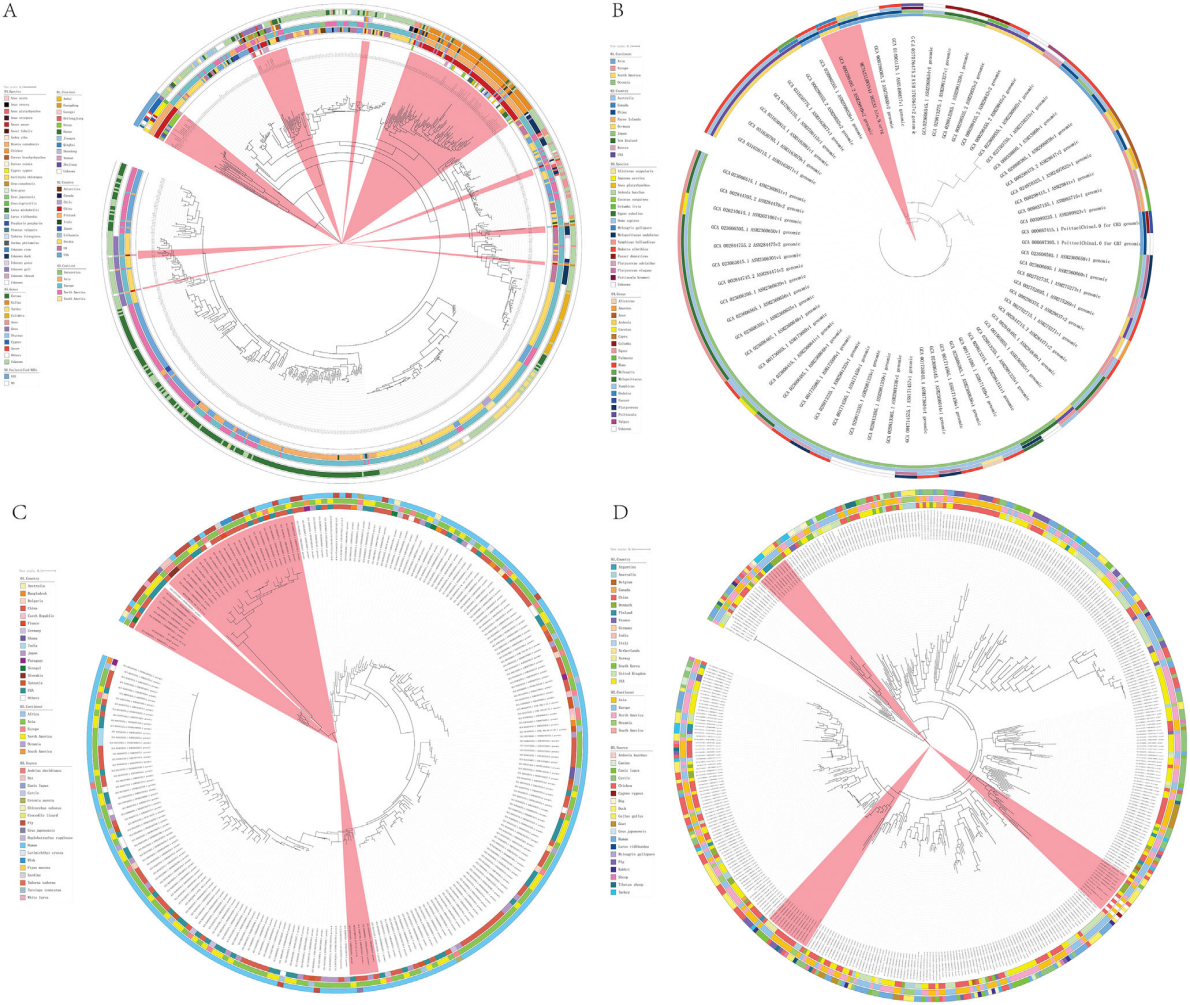
Phylogenetic analysis of four representative antimicrobial‐resistant zoonotic pathogens (*Campylobacter jejuni*, *Chlamydia psittaci*, *Morganella morganii*, and *Clostridium perfringens*). (A) Phylogenetic analysis of *Campylobacter jejuni* and novel *Campylobacter* spp. from 40 wild species globally. A total of 700 *Campylobacter jejuni* genomes from wild birds were downloaded and used in the comparative genomics analysis (Table S14). (B) Phylogenetic analysis of *Chlamydia psittaci* from wild birds, humans, and environmental sources globally. A total of 65 *Chlamydia psittaci* genomes were downloaded and used in the comparative genomics analysis (Table S15). (C) Phylogenetic analysis of *Morganella morganii* from wild birds, food animals, humans, and environmental sources globally. A total of 365 *Morganella morganii* were downloaded and used in the comparative genomics analysis (Table S16). (D) Phylogenetic analysis of *Clostridium perfringens* from wild birds, humans, and livestock globally. A total of 490 *Clostridium perfringens* were downloaded and used in the comparative genomics analysis (Table S17).

Then, we investigated the transmission dynamics, resistance, and virulent characteristics of zoonotic or opportunistic pathogens using MAGs and 1620 publicly available genomes of four representative species, including *Campylobacter jejuni*, *Chlamydia psittaci*, *Morganella morganii*, and *Clostridium perfringens* (Tables S14–S17). Phylogenetic analysis indicated that *Campylobacter jejuni* recovered from wild birds in China were relatively close to isolates from several regions, including the USA, Sweden, and the UK (Figure 5A). For example, *Campylobacter jejuni* recovered from *Larus ridibundus* in Yunnan, China, were clustered with those from the gull (unknown species) in Sweden. *Campylobacter jejuni* recovered from both *Grus japonensis* in Heilongjiang, Anser fabalis in Shandong, and *Grus nigricollis* in Yunnan, China, were clustered with those isolated from *Grus canadensis* in the USA. The phylogenetic tree shows that a *Chlamydia psittaci* genome recovered from *Ardeola bacchus* in Yunnan, China, in 2022 was clustered with a strain isolated from *Ondatra zibethica* in Canada (Figure 5B). Comparative genomic analysis revealed that one *Morganella morganii* recovered from *Tadorna tadorna* in Inner Mongolia, China, in 2016 was relatively close to isolates from *Chlorocebus sabaeus* in Senegal in 2019 and isolates from *Ondatra zibethica* in the USA in 1961 (Figure 5C). Moreover, one strain recovered from *Grus japonensis* in Heilongjiang, China, in 2023 was clustered with strains isolated from humans in the USA (Figure 5C). The opportunistic pathogen *Clostridium perfringens* recovered from *Larus ridibundus* in Kunming, China, in 2022 was clustered with strains isolated from *Gallus gallus* in Australia, in 2002 (Figure 5D). These results suggest that zoonotic pathogens or opportunistic pathogens may spread along with the cross‐national and intercontinental migrations of wild birds, posing potential risks to global public health. However, due to limited data, these results cannot explain whether these strains were spread through the migration of wild birds, which needs more data to support.

### 2.8 Host Prediction of the Migratory Virome and Analysis of Viral Auxiliary Metabolic Genes (vAMG)

**FIGURE 6:**
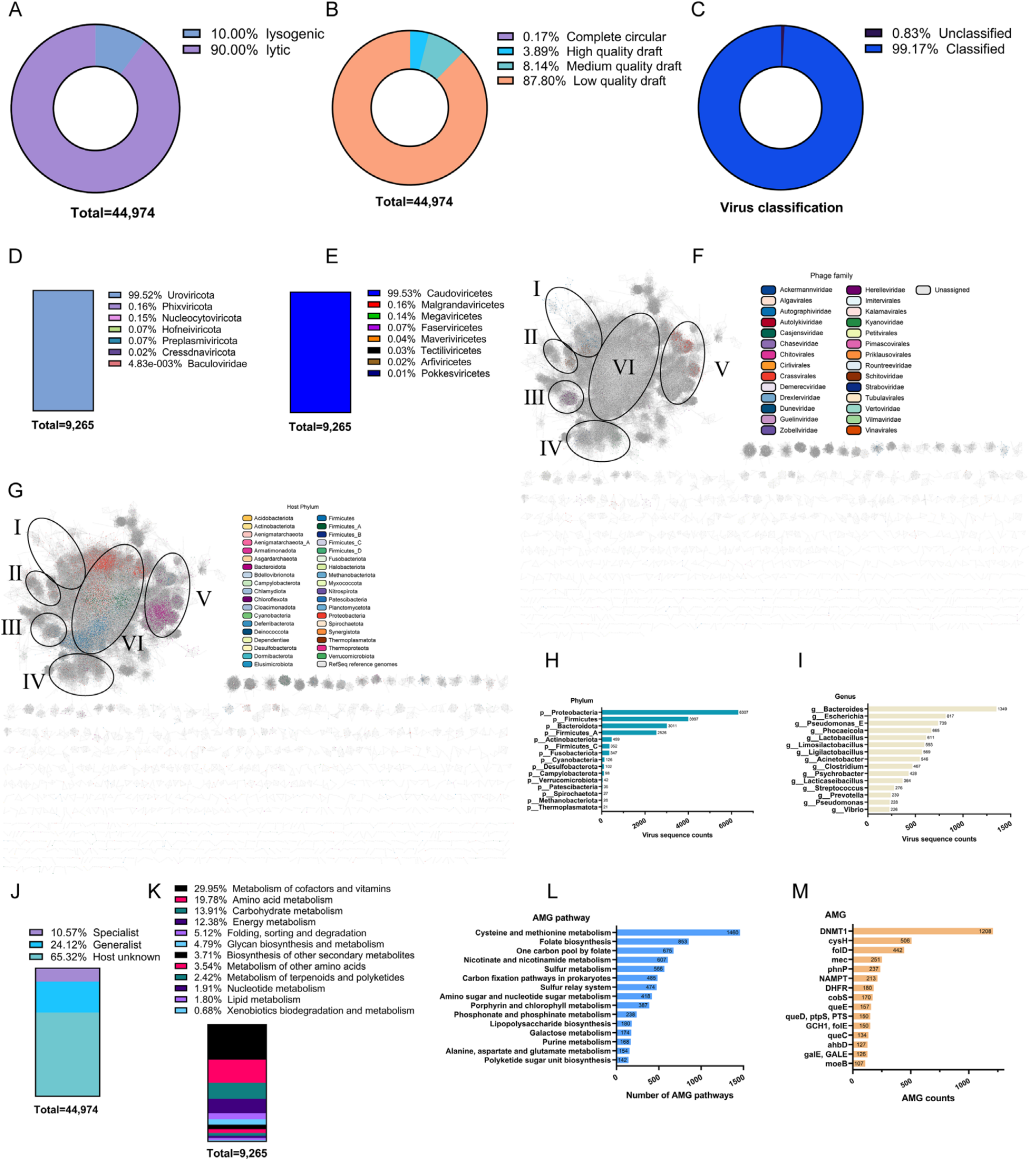
Taxonomic assignment and functional annotation of migratory virome catalog. (A) The prevalence of phage lifestyle in the migratory virome catalog. The lifestyle was predicted using VIBRANT. (B) Quality of the 44 974 viral genomes in the migratory virome catalog. Quality assessment was performed using VIBRANT. (C) The proportion of classified and unclassified virome. Virus classification is carried out using the geNomad. (D) Taxonomic assignment of prokaryotic virus genomes in migratory virome at the phylum level. (E) Taxonomic classification of migratory virome at the class level. (F) Gene‐sharing network of the vOTU obtained from vConTACT2 and visualized using Cytoscape v3.10.4 with the viral genomes being colored based on the family assignment. I: Autographiviridae and Schitoviridae; II: Schitoviridae; III: Herelleviridae; IV: Straboviridae and Kyanoviridae; V: Crassvirales; VI: Unassigned. (G) Gene‐sharing network of the vOTU with predicted host obtained from vConTACT2 and visualized using Cytoscape v3.10.4, and predicted hosts colored based on phyla. I: Proteobacteria and Campylobacterota; II: Proteobacteria; III: Proteobacteria and Firmicutes; IV: Proteobacteria, Firmicutes, and Bacteroidota; V: Bacteroidota; VI: Proteobacteria, Firmicutes_A and Firmicutes. (H) Overview of putative viral hosts grouped at the phylum level. I, Distribution of putative viral hosts grouped at the family level. (I) Distribution of putative viral hosts grouped at the genus level. (J) The diversity of the viruses as a function of their number of predicted hosts. The viruses could be divided into specialists (number of hosts = 1) and generalists (number of hosts > 1). (K) The metabolism categories of AMG pathways. (L) A barplot showing the categories of the top 15 most abundant AMG pathways. (M) A barplot showing the categories of the top 15 most abundant AMGs identified in the migratory virome catalog. See Table S19 for the detailed AMGs, full annotation of the final AMG‐carrying contigs, and AMG functional category annotation.

A total of 44 974 candidate non‐redundant viral genomes were recovered using state‐of‐the‐art bioinformatics approaches, combining VirSorter2 [44], CheckV [45], and VIBRANT [46]. According to the VIBRANT results, 90.00% of these genomes were putative lytic viruses, and the remaining genomes were putative lysogenic viruses (Figure 6A). Among these, 3.89% and 0.17% were high‐quality and complete circular genomes, respectively (Figure 6B). Using the GeNomad, these viral genomes were classified, and 99.17% of the DNA vOTUs could be classified into known clades (Figure 6C). At the phylum and class levels, 99.52% and 99.53n = % of these vOTUs belong to Uroviricota and Caudoviricetes, respectively (Figure 6D,E).

To better understand virus‒host linkages and gene sharing within these relationships, we generated a gene‐sharing network of all viral genomes with a host match and visualized it according to their taxonomy (Figure 6F) and their host phyla (Figure 6G). Based on the gene‐sharing network, most bird vOTUs were clustered into six groups (Figure 6F). Group VI had a broader host range among phyla, including Proteobacteria, Firmicutes_A, and Firmicutes. Groups III (mainly Herelleviridae) and V (mainly Crassvirales) contained more classified vOTUs than other groups. Groups II and V mainly infected Proteobacteria and Bacteroidota, respectively (Figure 6F). Of these putative hosts of viruses, a total of 71 and 17 506 bacteriophages were identified, respectively. These viruses were predicted to infect 339 phyla, 68 classes, 157 orders, 291 families, and 1094 genera (Figure 6H,I; Table S18). The dominant genus was *Bacteroides*, followed by *Escherichia* (Figure 6I). Overall, 10.57% and 24.12% of the 44 974 viruses with predicted hosts were classified as specialists and generalists, while the remaining viruses (65.32%) were predicted as host unknown (Figure 6J). The bacteriophages could infect 1035 known and 99 unknown genera of bacteria, and the archaeophages were predicted to infect 59 known and 12 unknown genera of archaea.

Among these 888 036 ORFs encoding in the viral genomes, a total of 107 069, 189 094, and 324 291 ORFs could be annotated using the KEGG, Pfam, and VOG databases, respectively. These vAMGs encode proteins involved in a variety of metabolic processes, for example, the metabolism of cofactors, vitamins, amino acids, carbohydrates, energy, glycan, nucleotides, lipid, and folding, sorting, and degradation (Figure 6K). A total of 1460 vAMGs were classified into cysteine and methionine metabolism genes, and 853 vAMGs were identified and belonged to folate biosynthesis (Figure 6L). A total of 4929 viral genomes carrying more than one vAMG (Table S19), the most prevalent vAMG encoding *DNMT1* was found in 1208 genomes, followed by *cysH* (506 genomes) and *folD* (442 genomes) (Figure 6M). Among these viral genomes, a total of 166 genomes carried at least one ARG, suggesting that ARGs are uncommon among wild bird virome. We found that rare ARGs were identified in phage genomes. A total of 9 ARGs, including *aadS* (*n* = 1) and *Escherichia coli emrE* (*n* = 8), were identified in these genomes.

### 2.9 The GPS Tracking System Helps to Assess the Migration of Microbial Communities and AMR Risk

**FIGURE 7:**
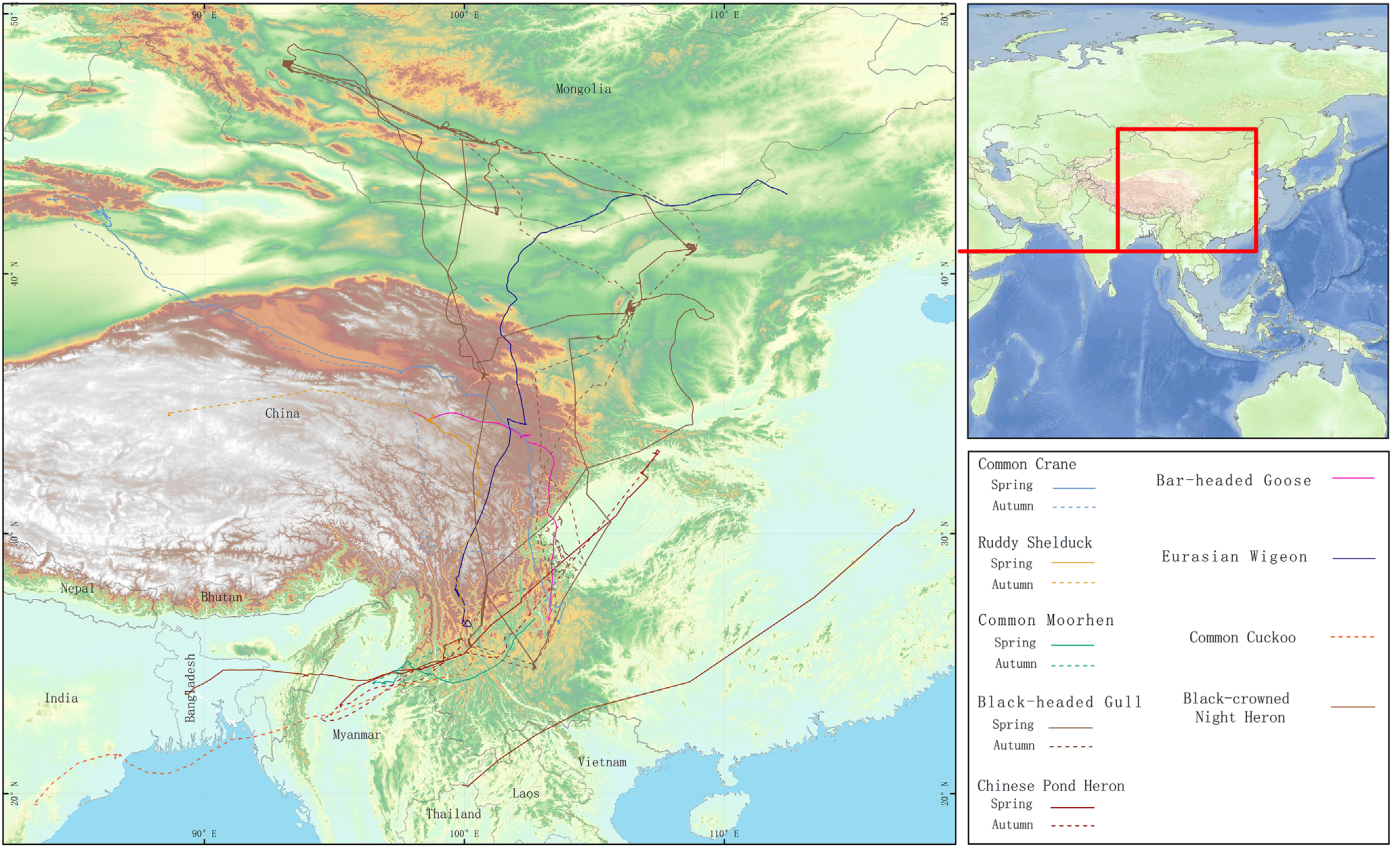
Migratory routes of wild birds across China's habitats. Migratory routes for a total of ten species of wild birds, including *Anas penelope* (Eurasian Wigeon), *Anser indicus* (Bar‐headed goose), *Ardeola bacchus* (Chinese Pond Heron), *Cuculus canorus* (common cuckoo), *Gallinula chloropus* (common moorhen), *Grus grus* (Common Crane), *Larus ridibundus* (Black‐headed Gull), *Nycticorax nycticorax* (black‐crowned night heron), *Tadorna ferruginea* (Ruddy Shelduck), and *Anas platyrhynchos*, were obtained using GPS. Note: The *Anas platyrhynchos* equipped with a GPS stayed in Jianhu Lake in Yunnan Province, China, and did not migrate to other countries or provinces in China. Therefore, the migratory route for *Anas platyrhynchos* was only in Yunnan and was not shown. The number of precise migratory movements: 100 888 in total; *Anas penelope*, *n* = 7291; *Anas platyrhynchos*: *n* = 22 189; *Anser indicus*: *n* = 2646; *Ardeola bacchus*: *n* = 4104; *Cuculus canorus*: *n* = 970; *Gallinula chloropus*: *n* = 6456; *Grus grus*: *n* = 17 870; *Larus ridibundus*: *n* = 20 175; *Nycticorax nycticorax*: *n* = 10 773; *Tadorna ferruginea*: *n* = 8414.

In addition, in several key habitats along important migratory bird corridors within Yunnan Province, China, we tracked ten migratory bird species and obtained their high‐resolution migration routes (Figure 7). A total of 100 888 location results from 10 migratory bird species tracked with 10 transmitters were recorded from January 2022 to November 2024. Of these records, the species with the most recorded individual location results is the *Anas platyrhynchos* (*n* = 22 189), followed by *Larus ridibundus* (*n* = 20 175), *Grus grus* (*n* = 17 870), and *Nycticorax nycticorax* (*n* = 10 773). These migratory routes showed that waterbirds that winter in Yunnan migrated to regions such as Russia and Mongolia during spring migration and summered at conjunction regions between Russia, Mongolia, and China (Figure 7). During autumn migration, some species like the *Common cuckoo* flew to India, while the night heron passed through Myanmar and Thailand before wintering in Bangladesh, South Asia (Figure 7). Domestically, these migratory birds mainly migrated along the Central Asian migratory corridor, with summer breeding sites including Xinjiang, Qinghai, Xizang, Gansu, Inner Mongolia, Sichuan, and Anhui province (Figure 7). Additionally, some species, like the *Eurasian wigeon*, migrated to Heilongjiang in northeastern China during summer (Figure 7). Important stopover sites along the migration routes are located in central regions of the Yellow River basin, such as Gansu, Ningxia, and Inner Mongolia. Some species, like the Black‐headed gull, exhibited strong stability in their wintering site selection, returning to the same location for several consecutive years (Figure 7). Overall, these results reveal that wild birds carried (opportunistic) pathogens and ARGs equipped with GPS transmitters could provide high‐resolution maps of zoonotic and AMR spread risk, from the local to regional and international regions, conversely, so on.

## 3 Discussion

Here, we present, to the best of our knowledge, the first migratory bacterial and archaeal microbiome and gene catalog for wild birds across China's habitats, comprising 5823 MAGs, 74 million proteins, 13 072 plasmid sequences, and 44 974 viral genomes from 340 bird metagenomes. Migratory birds have been considered as global AMR and zoonotic pathogen sentinels [2, 6]. The free long‐distance migration behavior of birds provides wings for AMR and zoonotic pathogens, posing a significant challenge to global public health. Nevertheless, MAGs representing 959 known and 1709 novel species were recovered from these migratory microbiomes, comparable to similar efforts from glacier microbiomes with extreme environments [15]. Wild birds harbor diverse and novel microbial populations, with 49.10% of the MAGs representing novel species. Overall, this resource of 5823 MAGs and 44 974 viral genomes represents the greatest effort to date on behalf of the coverage of bacterial, archaeal, and viral genomic diversity from birds across China's habitats. The diversity of the microbial community in the catalog is further confirmed by the large proportion of protein sequences with novel functions. Therefore, the continuous work of recovering genomes of novel candidate species and strain‐level representatives from metagenomes will further enhance the value of these datasets.

The prediction of the metabolic functions of gut microbiomes in different wild bird species may reflect the differences in living habitats and specific environmental adaptations [2]. Our results suggested that the abundance of BGCs synthesizing RiPP clusters was the most diverse in wild birds. Notably, BGCs synthesizing PKS/NRPS Hybrids and other clusters mainly originated from uSGBs. In addition, birds in different habitats may contribute to the differences in the metabolic functions observed among different species. Further metagenomic studies on both the migratory birds and the resident birds can provide more insights into the functions of bird microbiomes. Our study provides a valuable catalog for the integration and unification of global wild bird metagenomic data and timely sharing in the future.

Wild birds were considered sentinels for monitoring zoonotic pathogens [6, 47] and ARGs [3, 4, 86]. In this study, we identified 1814 known ARGs using read mapping methods and blast search for MAGs from the bird microbiome, indicating that migratory bird microbiomes are a huge ARG reservoir. Similar to previous studies [2, 3, 48], multiple clinically important ARGs, including carbapenem, colistin, and oxazolidinone resistance, were identified in wild birds.

Genomic and big data‐driven studies in modern microbiology not only help us improve the recognition of uncultured microorganisms, but also reveal the important role of non‐cellular genetic elements such as viruses (phage) and plasmids, providing novel insights into ecological, AMR, and evolutionary studies [49]. To our knowledge, no studies have explored the role of plasmidome and virome (all phages) in the transmission of AMR in migratory pathogen repositories. The emergence of bioinformatics tools (VirSorter2 [44], CheckV [45], VIBRANT [46] vConTACT2 [46], and GeNomad [50]) to recover viral sequences in metagenomic assemblies significantly enhances our understanding of new viral genomes in the human gut [51], chicken [16], rumen [52], goat [18], and soils [53].

Today, microbiologists, genomics, and computational scientists are struggling to overcome the difficulty of mining plasmid sequences from vast amounts of metagenomic data. Recently, the emergence of novel bioinformatics tools such as plasmid recognition, classification, and annotation [38, 39, 40, 50, 54] has been applied to human gut microbiome studies and recovered thousands of plasmids [55, 56]. However, our understanding of plasmids in metagenomes from wild birds is still limited by methodological challenges and resources for wild bird species diversity. Therefore, so far, there is still a gap in the migratory microbiome reference catalog from wild birds in China. However, our understanding of plasmid diversity is still scarce in the bird gut due to methodological limitations.

In this study, a valuable plasmid catalog was built using a total of 13 072 contigs recovered from 340 metagenomes. The association analysis indicated most of the plasmid sequences connected with the potential host within the Proteobacteria phylum, with a high frequency of detection of resistance genes and the presence of a shuttle event. These results highlight the significance of MBGG as a plasmid resource, especially given the current scarcity of studies on plasmids in the microbial communities of wild birds. Overall, all metagenome‐assembled genomes, plasmid sequences, viral genomes, BGCs, ARGs, VFs, and non‐redundant protein‐coding genes can be available in Figshare, https://figshare.com/s/8699370b85d1875209b2.

Previous studies [10, 11, 12, 13, 14, 15, 16, 17, 18, 19, 20, 21, 22, 23, 24, 25, 26, 27] enlarged our understanding of functional genes (for example, ARGs, antimicrobial peptides, and metabolism) of MAGs from the human microbiome, ocean, glacier, chickens, goat, pigs, ruminants, horse, wild animals, pandas, soil, and environmental samples. However, these datasets did not involve wild birds across China's habitats. In this work, a valuable comprehensive resource by integrating the metagenomic data of 50 species of wild birds provides lots of uncultivated species, BGCs, CAZymes, and ARGs, greatly enhancing our understanding of taxonomic novelty, unique BGCs, CAZyme diversity, plasmid diversity, and AMR patterns. To our knowledge, this is the first genomic catalog of metagenomes from wild birds worldwide.

Previous studies [57, 58, 59] have shown that using GPS to track the high‐resolution migration locations of wild birds, combined with land cover information, can contribute to predicting pathogen risk maps and critical habitats. Analysis of 10 GPS tracks results in 107 888 locations collected from 2022 to 2024 demonstrated that 10 bird species visited different habitats in their migration routes. These results provide a critical warning for national biosecurity management and the use of extensive multispecies GPS tracking to highlight potential high‐risk MDR/XDR clones, ARGs, and highly pathogenic microorganism spread locations, and to more effectively respond.

This study has several limitations. Although this is one of the largest bacterial microbiome studies of migratory birds across China's habitats to date, it was based on opportunistic sampling in some specific geographical locations and therefore cannot be regarded as a national active monitoring behavior. Despite our best efforts in sampling, in some cases, the sample size was very small, and the coverage of bird species (only 50 species) was not wide enough. In addition, due to the short read sequencing and assembly length limit, draft genomes of MAGs and viral genomes were obtained, while the quality and integrity of MAGs and viral genomes still need to be improved in the future. Similarly, due to the short read sequencing approach, the identification of ARGs or fragments, the genetic environment of ARGs could not be investigated. Furthermore, although we constructed a non‐redundant catalog composed of 20 million ORFs and mined the diversity and abundance of secondary metabolite BGCs, ARGs, VFs, and CAZyme‐encoding genes, many genes remain to be explored, among which many functions are unknown. Finally, although some metagenomic studies using in‐depth analysis have been used to explore the plasmidome in the human gut [55, 56] and hospital effluents [85], only a small number of plasmids were recovered from bird microbiomes, limited by methodological challenges.

In conclusion, the multi‑kingdom MBGG catalog provides a database for mining and comparison of wild bird microbiomes at the bacterial and viral genome and gene levels. Here, we displayed its value in the mine of microbial and phage genomes, secondary metabolites BGCs, ARGs, and virulence genes diversity, and public health biosafety. We assume that this catalog will form the basis of a global, comprehensive wild bird microbiome data resource. This work fills a significant knowledge gap in the exploration of the diversity and function of the gut microbiomes in wild birds across China's habitats, providing a valuable resource model for the assessment of the migration impact of AMR and zoonotic pathogens brought about by bird migration.

## 4 Methods

### 4.1 Sample Collection, DNA Extraction, and Metagenomic Sequencing

A total of 593 samples were collected between September 2016 and December 2023 from bird wintering/stopover sites in 11 different provincial administrative districts (including Swan Lake wetland in Sanmenxia, Henan; Dongting Lake wetland in Hunan; Jianhu Lake wetland, Dianchi Lake wetland, and the Black‐necked Crane Nature Reserve of Huize in Yunnan; Poyang Lake wetland in Jiangxi; Wetland National Nature Reserve in Jiangsu; National Wetland Park in Zhejiang; Wetland Park in Dongying, Shandong; Genggahai Lake wetland and Qinghai Lake wetland in Qinghai; Lhasa in Tibet; Ordos in Inner Mongolia; and Zhalong National Nature Reserve and Longfeng Wetland Nature Reserve in Heilongjiang) in China (Figure 1A; Table S1). Among them, a total of 448 new samples were collected from different individual birds and associated ecological niches. All samples were stored in sterile EP tubes and transported to the laboratory by dry ice for further processing. The Ethical Approval for Research Involving Animals (number: IACUC‐OE‐2021‐08‐004) was granted by the Institutional Animal Care and Use Committee, Kunming Institute of Zoology, Chinese Academy of Sciences. Total DNA was extracted for each sample using the PowerSoil DNA isolation kit (Qiagen) following the manufacturer's instructions. All DNA was stored at −80°C. Illumina libraries were prepared using the KAPA Hyper Prep Kit (Roche) following the manufacturer's instructions. A total of 195 new libraries were built and then sequenced using the Illumina NovaSeq 6000 platform PE150 with 150 bp paired‐end reads at the Microbial Genome Research Center, Institute of Microbiology, Chinese Academy of Sciences. In addition, a total of 21 wild bird metagenomic samples generated in a previous study [60] and 124 bird metagenomes described in our previous study [2], which mainly focused on the abundance and diversity of ARGs and microbial community, were also included and analyzed.

### 4.2 Quality Control and Assembly

Quality control (such as removing low‐quality sequences, PCR duplication, and residual adapter sequences) was performed using the fastp v0.23.4 (https://github.com/OpenGene/fastp) with the parameters “‐W 4 ‐M 20 ‐n 5 ‐c ‐l 50 ‐w 3” [61]. Bowtie2 v2.3.4 (https://sourceforge.net/projects/bowtie‐bio/files/bowtie2/2.3.0/) [62] was used to filter out the host genomic DNA sequence (chicken, GCF_000002315.6_GRCg6a; human, hg37dec_v0.1; pig, no.NC_010443; cattle, GCA_002263795; goat, GCF_001704415.2_ARS1.2; Rnor_6, GCF_000001895.5_Rnor_6.; sheep, GCF_002742125.1_Oar_rambouillet_v1.0; wild‐yak, GCF_000298355.1_BosGru_v2.0; dog, GCF_000002285.5_Dog10K_Boxer_Tasha; mouse, mouse_C57BL_6NJ; Ochotona curzoniae, GCF_017591425.1_NIBS_Ocur_1.0). Then, the remaining high‐quality paired clean reads were assembled into contigs for each sample using MEGAHIT v1.2.9 (https://github.com/voutcn/megahit) [63] with the default k‐mer parameters, and only contigs ≥500 bp were retained for further use. Assembly quality was checked using Quast v5.3.0 (https://github.com/ablab/quast) [64], and statistical data were available in Table S1.

### 4.3 Metagenomic Binning and Genome Quality Evaluation

For metagenomic binning analysis, three different binning tools, including MetaBAT2 v.2.12.1 [65], Maxbin2 v2.2.7 [66], and Concoct v.1.1.0 [67] in the metaWRAP v1.3.2 pipeline (https://github.com/bxlab/metaWRAP) [68] were used. The resulting MAGs (or bins) were refined using the bin_refinement module of metaWRAP with the parameters “‐c 50 ‐x 10” [68]. The reassemble_bins module of the metaWRAP was used to reassemble these bins to improve the quality [63]. The completeness and contamination of MAG were evaluated by CheckM v1.2.2 (https://github.com/Ecogenomics/CheckM) [69]. The low‐quality bins with contamination > 10% and completeness < 50% were removed according to CheckM v1.2.2 [69], and a total of 6166 bins were obtained. Then, MAGpurify v2.1.2 (https://github.com/snayfach/MAGpurify) was used to remove contamination sequences with default modules and parameters [12]. In addition, CheckM2 v1.1.0 (https://github.com/chklovski/CheckM2) was reused on these refined bins to calculate the contamination, completeness, and strain heterogeneity [70]. The quality score of at least medium quality was considered valid MAGs [14, 70]; the low‐quality bins were removed in the following analysis.

### 4.4 Construction and Annotation of the MBGG Catalog

The genome taxonomy database toolkit (GTDB‐Tk v2.3.2, https://github.com/Ecogenomics/GTDBTk) was used to assign taxonomy to the MAGs with the classify_wf module (default parameters) [36]. Finally, a total of 5823 bacterial and archaeal bins met the criterion of medium‐ or high‐quality scores and were obtained as the migratory MAG catalog. The fastANI v1.1 (https://github.com/ParBLiSS/FastANI) was used to calculate the ANI between bins generated in this study; ANI < 0.95 were designated as novel species. The CompareM v0.1.2 (https://github.com/dparks1134/CompareM) was used to calculate the average amino acid identity (AAI) values between bins. The dRep v3.4.2 (https://github.com/MrOlm/drep) with the parameters “‐p 16 ‐d ‐comp 50 ‐con 5 ‐nc 0.25 ‐pa 0.9 ‐sa 0.99” was used to dereplicate these redundant bins [71]. The 5823 genomes were dereplicated into 2987 non‐redundant MAGs. The PhyloPhlAn v3.1.1 (https://github.com/biobakery/phylophlan) was used to construct the phylogenetic tree, including bacterial and archaeal bins [72]. ORFs of all contigs were predicted by Prodigal v2.6.3 (https://github.com/hyattpd/Prodigal) [73] and were dereplicated at 90% coverage and 95% identity using cd‐hit v4.8.1 with parameters “‐c 0.95 ‐aS 0.9 ‐G 0 ‐s 0.8 ‐n 5 ‐M 20000 ‐g 1 ‐d 0 ‐T 64” (https://github.com/weizhongli/cdhit) [74]. Then, a total of 20 062 410 non‐redundant genes using the criteria of identity 95% and overlap 90% were obtained from 74 076 453 ORFs. In addition, a total of 27 515 964 ORFs (identity 100%) and 12 562 894 ORFs (identity 50%) were obtained, respectively.

The migratory MAG catalog was functionally annotated using Distilled and Refined Annotation of Metabolism (DRAM v1.5.0, https://github.com/shafferm/DRAM) [75] with default parameters and Prokka v1.14.5 (https://github.com/tseemann/prokka) with parameters “–centre X –compliant –cpus 96 –kingdom Bacteria –metagenome” [76]. The eggNOG‐mapper v2.1.13 (https://github.com/eggnogdb/eggnog‐mapper) with parameters “‐m diamond, e‐value threshold 0.001” was used to annotate the non‐redundant gene catalog [37]. The KEGG functional orthologs database (https://www.genome.jp/kegg/), Cluster of Orthologous Groups categories (COG) database (https://www.ncbi.nlm.nih.gov/research/COG), and the carbohydrate‐active enzymes (CAZy, https://www.cazy.org/) database were integrated with both DRAM [75] and eggNOG‐Mapper v2.1.13 [37]. ARGs were annotated using RGI v6.0.3 (https://github.com/arpcard/rgi) [33] against the CARD database with parameters “Cut_Off: Strict, Perfect; Best_Identities≥80” [88, 89] and ResFinder v4.1 (https://bitbucket.org/genomicepidemiology/resfinder/src/master/) with parameters “‐l 0.6 ‐t 0.8” against the ResFinder (https://bitbucket.org/genomicepidemiology/resfinder_db/src) and PointFinder database (https://bitbucket.org/genomicepidemiology/pointfinder_db/src/master/) [35]. The virulent factors were identified using ABRicate v1.0.1 (https://github.com/tseemann/abricate/, accessed at April 19th, 2024) with default parameters “Minimum DNA %identity = 80, Minimum DNA %coverage = 80” against the Virulence Factor Database (VFDB) [41, 87, 90] and through the MetaVF toolkit with parameters “‐m draft ‐c 10 ‐ti 90 ‐tc 80” (https://github.com/Wanting‐Dong/MetaVF_toolkit) against the expanded Virulence Factor database (VFDB 2.0, https://github.com/Wanting‐Dong/MetaVF_toolkit/tree/main/databases, accessed at November 4th, 2024) [42]. In addition, the MobileElementFinder v1.1.2 (https://bitbucket.org/mhkj/mge_finder/src/master/, accessed on August 21st, 2023) with parameters “–min‐coverage 0.95, –min‐coverage 0.9” was used to identify MGEs, including insertion, transposon, integrative conjugative elements, and integrative mobilizable elements in all MAGs [77]. The ORFs that failed to be against any of these databases were designated as novel candidate functional genes.

### 4.5 Secondary Metabolite Biosynthetic Gene Clusters (BGCs) Analysis

To explore the functional potentials of the migratory microbiomes, the AntiSMASH v7.1.0.1 (https://github.com/antismash/antismash) was used to identify secondary metabolite BGCs from contigs more than 3 kb in the MAGs with default parameters [78]. These AntiSMASH‐generated specific BGC families were classified into eight different classes: type I polyketide synthases (PKS), other PKSs, non‐ribosomal peptide synthetases (NRPS), PKS/NRPS hybrids, RiPPs, Terpene, Saccharides, and others according to the biosynthetic gene similarity clustering and prospecting engine (BiG‐SCAPE v2.0.0 https://github.com/medema‐group/BiG‐SCAPE) [79].

### 4.6 Diversity and Abundance of Migratory Microbiome and Potential Pathogens

The taxonomic abundance profiles of the migratory microbiomes were annotated for high‐quality metagenomic reads using the MetaPhlAn4 v4.1.1 (https://github.com/biobakery/MetaPhlAn, accessed in May 2025) with default parameters [43]. According to the National Pathogen Resource Center in China (https://www.nprc.org.cn/) and the lists of pathogenic microorganisms that are transmitted between humans (http://www.nhc.gov.cn/qjjys/s7948/202308/b6b51d792d394fbea175e4c8094dc87e.shtml), the diversity of human pathogenic microorganisms, opportunistic pathogens, or zoonotic pathogens in migratory microbiomes was summarized and analyzed.

### 4.7 Diversity and Abundance of Known ARGs and MGEs

We first focused on the known ARGs, and the abundance profile of ARGs was obtained using the ARGs‐OAP3 v3.1.1 with parameters “–e 1e‐7 –id 90 –qcov 75 –length 25” (https://github.com/alienzj/args_oap) [32]. The risk of ARGs was also classified into four grades of risk levels, according to whether they meet the three criteria: presence in the human living environment, association with human pathogens, and mobility, in previous reports [80]. In addition, the abundance profile of MGEs was obtained using the BBmap (https://github.com/BioInfoTools/BBMap) by mapping reads to an MGE database generated in previous reports [81].

### 4.8 Reconstruction and Annotation of Plasmidome

First, the Sequence Contents‐Aware Plasmid Peeler (SCAPP v0.1.4, https://github.com/Shamir‐Lab/SCAPP) was used to identify plasmid sequences from all migratory metagenomic sequences [38]. Second, the MOB‐suite v3.1.9 (https://github.com/phac‐nml/mob‐suite) was used to type plasmids into mobilizable, conjugative, and non‐mobilizable classes by blasting for plasmid markers, including mobilization proteins (relaxase), replicase genes (rep), origin of transfer (oriT) sites, and the mate‐pair formation system (MPF) genes [40].

### 4.9 Recovery, Host Prediction, and Functional Annotation of the Virome

Viral sequences were identified through the modified viral sequence identification SOP (https://doi.org/10.17504/protocols.io.bwm5pc86), according to the previous report [16, 52]. Briefly, we first identified viral sequences using VirSorter2 v2.2.4 (https://bitbucket.org/MAVERICLab/virsorter2) [44] with parameters “–includegroups dsDNAphage, ssDNA, ‐min‐score 0.8” and ran the CheckV v1.0.1 (https://bitbucket.org/berkeleylab/checkv/src/master/) [45] to remove host sequences flanking prophages. Then, we run the VirSorter2 v2.2.4 again to verify the viral sequence and generate “affi‐contigs.tab” files. In total, 47 452 putative viral genomes > 5 kb were identified. The putative viral genomes were clustered into species‐level viral operational taxonomic units (vOTUs) at 95% ANI and 85% alignment fraction (AF) of the shorter sequence using script aniclust.py with the options: “–min_ani 95 –min_tcov 85” from the CheckV repository [45]. The VIBRANT v1.2.1 (https://github.com/AnantharamanLab/VIBRANT, option: –virome) [46] was also used to verify these viral sequences. A total of 44 974 genomes and 44 193 vOTUs were identified. The putative viral genomes with no gene showing the best hit to prokaryotic viruses and originating from eukaryotic viruses were removed. The completeness of the vOTUs was estimated using both CheckV v 0.8.1 [45] and VIBRANT v1.2.1 [46]. Only the putative viral sequences identified by both VirSorter2 v2.2.4, CheckV v1.0.1, and VIBRANT v1.2.1 simultaneously (score≥0.8, contig>5 kb) were listed in the migratory virome catalog and kept for taxonomic annotation and host prediction according to previous reports [16, 46, 52]. A gene sharing network was built using vConTACT2 v0.11.3 (https://bitbucket.org/MAVERICLab/vcontact2/src/master/) [82].

The GeNomad v1.7.1 (https://github.com/apcamargo/genomad/) 200 000 marker protein profiles were used for taxonomic annotation of the putative viral genomes and plasmid sequences [50]. The iPHoP v1.3.3 (https://bitbucket.org/srouxjgi/iphop/src/main/, –min_score 90) [83] integrated phage‐based method (RaFAH) and host‐based approach (blast, CRISPR, PHP, WIsH, and VirHostMatcher) was used to predict the probable hosts of the migratory virome (phage). The DRAMv [75] and VIBRANT v1.2.1 [46] were used to identify vAMGs from phage genomes. The lifestyles and quality of migratory virome were predicted using the VIBRANT v1.2.1 [46]. In addition, the functional genes against the protein families database (Pfam, http://pfam.xfam.org/), carbohydrate‐active enzyme (CAZyme, http://www.cazy.org/), and virus orthologous groups (VOG, https://vogdb.org/) were obtained and kept for downstream analysis.

### 4.10 Global Positioning System (GPS) Tracking System

Migratory birds are known to transmit pathogens and AMR genes over long distances via migratory routes, so we used a 3‐year GPS tracking dataset to evaluate wild birds' movements relative to the spread of AMR pathogens and ARGs across China since 2022. The migratory routes of 10 representative bird species including *Anas penelope* (Eurasian Wigeon), *Anser indicus* (Bar‐headed goose), *Ardeola bacchus* (Chinese Pond Heron), *Cuculus canorus* (Common cuckoo), *Gallinula chloropus* (Common moorhen), *Grus grus* (Common Crane), *Larus ridibundus* (Black‐headed Gull), *Nycticorax nycticorax* (Black‐crowned night heron), *Tadorna ferruginea* (Ruddy Shelduck), and *Anas platyrhynchos* were tracked using commercially available global positioning system (GPS) transmitters attached to 10 birds covering 10 species in Yunnan, China, from January 2022 to November 2024. We have acquired 100 888 locations from 10 individuals of 10 species of wild birds (Table S1) moving along the flyways. A detailed high‐resolution migration route, including a total of 100 888 location results, was available in “https://figshare.com/s/8699370b85d1875209b2”.

### 4.11 Public Dataset Download

A total of 1620 bacterial genomes, including 700 *Campylobacter jejuni*, 490 *Clostridium perfringens*, 365 *Morganella morganii*, and 65 *Chlamydia psittaci* genomes, were downloaded for comparative genomic analyses (for details, see Tables S13–S16). In addition, 21 public metagenomic datasets were downloaded for metagenome‐assembled genomes, secondary metabolite biosynthetic gene clusters, antibiotic resistance genes, virulence factors, and virome analysis (for details, see Table S1).

### 4.12 Statistical Analyses and Visualization

The phylogenetic tree was visualized through the interactive tree of life (iTOL v7.0, https://itol.embl.de/) [84]. Multiple comparisons (including Kruskal‐Wallis test and Mann‐Whitney U test) were performed using Prism v9.0. Statistical significance was considered at *P* < 0.05. The gene sharing network was visualized using Cytoscape v3.10.4 (https://cytoscape.org/).

## Author Contributions

Y.W. and G.F.G. conceived and designed the project. Y.W., H.W., M.Q., and C.Z. collected samples and epidemiological data. H.W. performed the global positioning system (GPS) for migratory birds and collected the GPS data. Y.W. organized the metagenomic sequencing, downloaded the public dataset, performed bioinformatics analysis, statistical analysis, created the figures and tables, and drafted the original manuscript. Z.X., Y.P., C.Z., J.W., S.M., N.L., X.X., Y.B., and B.Z. provided invaluable insights into the analysis. All authors discussed and interpreted the data and contributed to the manuscript. All authors read and approved the submitted version.

## Conflicts of Interest

The authors declare no conflicts of interest.

## Code Availability Statement

The source code used for “A genomic catalog of migratory microbiomes from wild birds across China's habitats” analysis in this study is available at https://github.com/NANYW123/migratory‐bird‐microbiome‐catalog‐MBGG.git.

## Supporting information




**Supporting File 1**: advs74581‐sup‐0001‐SuppMat.docx.

**Supporting File 2**: advs74581‐sup‐0002‐TableS1‐S19.xlsx.

## Data Availability

All sequencing data generated in this study have been deposited to NCBI under BioProject number PRJNA1379261 (*n* = 195), and publicly available metagenomes were downloaded from BioProject numbers PRJNA556790 (*n* = 103), PRJNA563508 (*n* = 21), and PRJNA890321 (*n* = 21). BioSamples for each sample were available in Table S1. All metagenome‐assembled genomes (bacterial and archaeal MAGs), plasmid sequences, viral sequences, BGCs, ARGs, VFs, and non‐redundant gene catalog generated in the present study are available in the Figshare repository with the identifiers “https://figshare.com/s/8699370b85d1875209b2”, DOI: https://doi.org/10.6084/m9.figshare.30931052. Any remaining information can be obtained by contacting the corresponding author upon reasonable request.
