# Supplementary material for: A Genomic Catalog of Migratory Microbiomes from Wild Birds across China's Habitats
Source: Adv Sci (Weinh). 2026 Feb 26;13(26):e74581. doi: 10.1002/advs.74581 (PMC13159146; doi:10.1002/advs.74581)
Supplement: Supplementary file 1 — Supporting File 1: advs74581‐sup‐0001‐SuppMat.docx. [file ADVS-13-e74581-s002.docx]

A genomic catalog of migratory microbiomes from wild birds across China’s habitats

Yanan Wang^1,2,3^^,9,11,12,*^, Heqi Wu^4,11^, Mengqi Qu^1^, Chunge Zhang^2,5^, Ziqian Xu^6^, Yuhang Pei^1^, Chaochao Zhao^1^, Jiangtao Wang^1^, Sufang Ma^2^, Na Lyu^2^, Xuebin Xu^7^, Yuhai Bi^2,5,8^, Baoli Zhu^2,5,9^, George F. Gao^2,5,6,8,9,10^

^1^International Joint Research Center of National Animal Immunology, College of Veterinary Medicine, Henan Agricultural University, Zhengzhou, 450046, China.

^2^CAS Key Laboratory of Pathogen Microbiology and Immunology, Institute of Microbiology, Chinese Academy of Sciences (CAS), Beijing 100101, China.

^3^Longhu Laboratory of Advanced Immunology, Zhengzhou, 450046, China.

^4^State Key Laboratory of Genetic Evolution & Animal Models, Kunming Institute of Zoology, Chinese Academy of Sciences, Kunming 650201, China

^5^University of Chinese Academy of Sciences, Beijing 100049, China.

^6^National Institute for Viral Disease Control and Prevention, Chinese Center for Disease Control and Prevention, Beijing 102206, China.

^7^Division of Pathogen Testing and Analysis, Shanghai Municipal Center for Disease Control and Prevention, Shanghai, 201107, China.

^8^Center for Influenza Research and Early-Warning, CAS-TWAS Center of Excellence for Emerging Infectious Diseases, Chinese Academy of Sciences, 100101 Beijing, China.

^9^Beijing Key Laboratory of Antimicrobial-Resistant Pathogen Microbiology and AI-Empowered Containment, Institute of Microbiology, Chinese Academy of Sciences (CAS), Beijing 100101, China.

^10^Chinese Center for Disease Control and Prevention, Beijing, 102206, China.

^11^These authors contributed equally to this work.

^12^Lead Contact.

^*^E-mail: [wangyanan1001@henau.edu.cn](mailto:wangyanan1001@henau.edu.cn) (YNW)


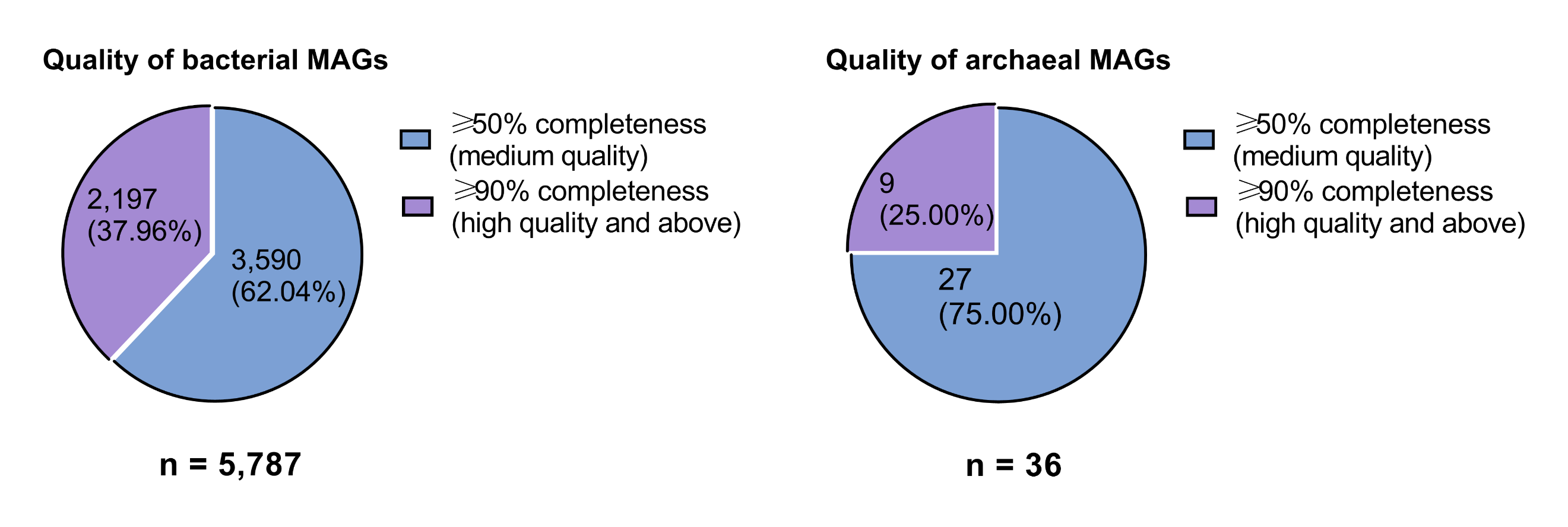


Supplementary Fig. 1. Quality of the 5,787 bacterial and 36 archaeal genomes.


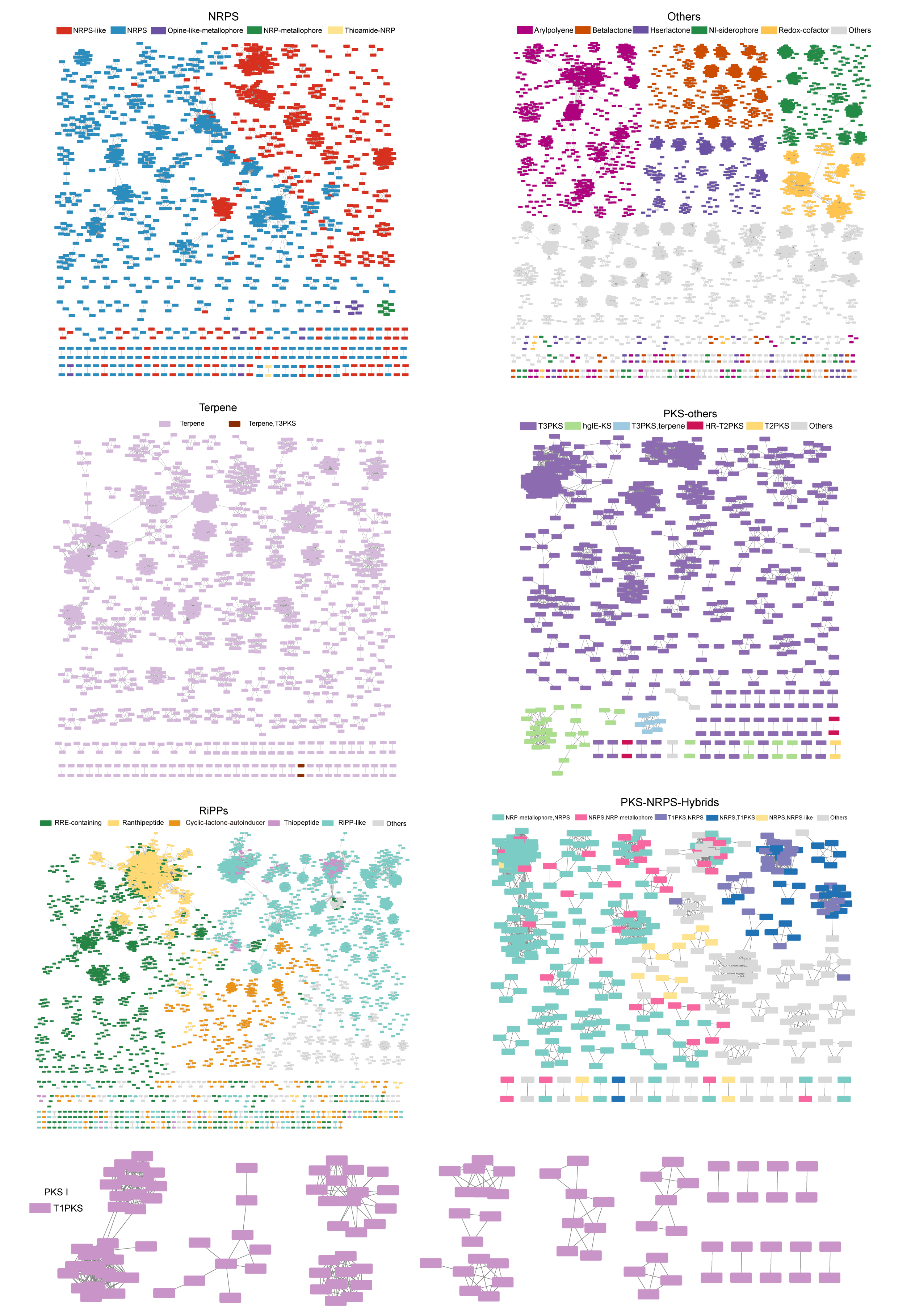
Supplementary Fig. 2. Sequence similarity networks of BGCs using the BiG-SCAPE method. A detail of the BiG-SCAPE network for different BGC classes sequences.


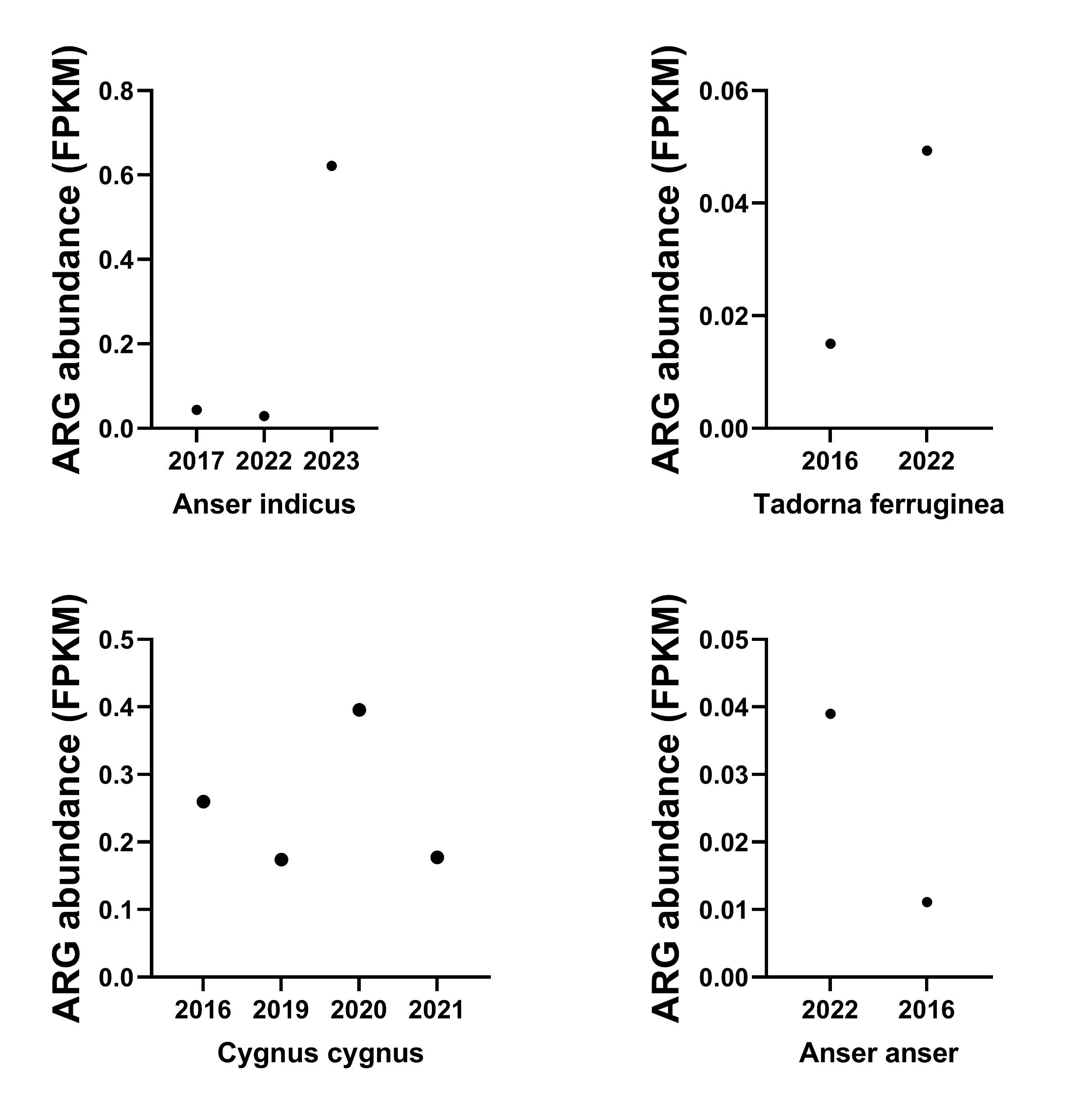


Supplementary Fig.3. Plots showing the average AMR level per sample among *Anser indicus*, *Tadorna ferruginea*, *Cygnus cygnus*, and *Anser anser* in different sampling periods.


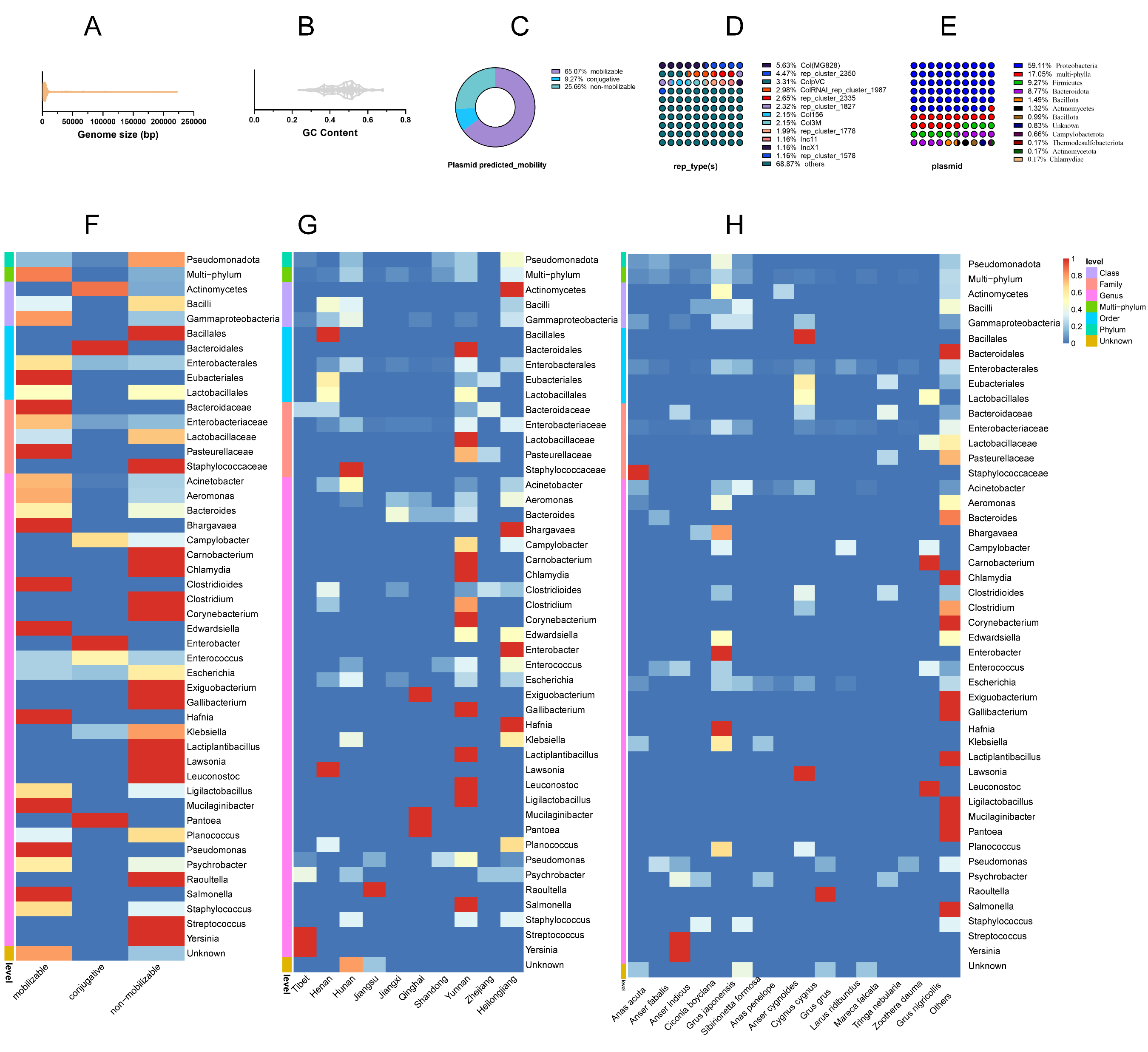


Supplementary Fig. 4. Distribution and association with bird gut plasmids and bacterial hosts.

A, Distribution of genome size of plasmids in the migratory microbiomes. B, Distribution of GC contents of plasmids in the migratory microbiomes. C, Mobility of recovered plasmids in the migratory microbiomes. D, Distribution of replicon types in the migratory microbiomes. E, The number of plasmids associated with different bacterial hosts, grouped by phylum. F, Heatmap showing the richness of recovered plasmids in different bacterial hosts. G, Distribution of recovered plasmids in different sampling locations. H, Heatmap showing the richness of recovered plasmids in different bird species.


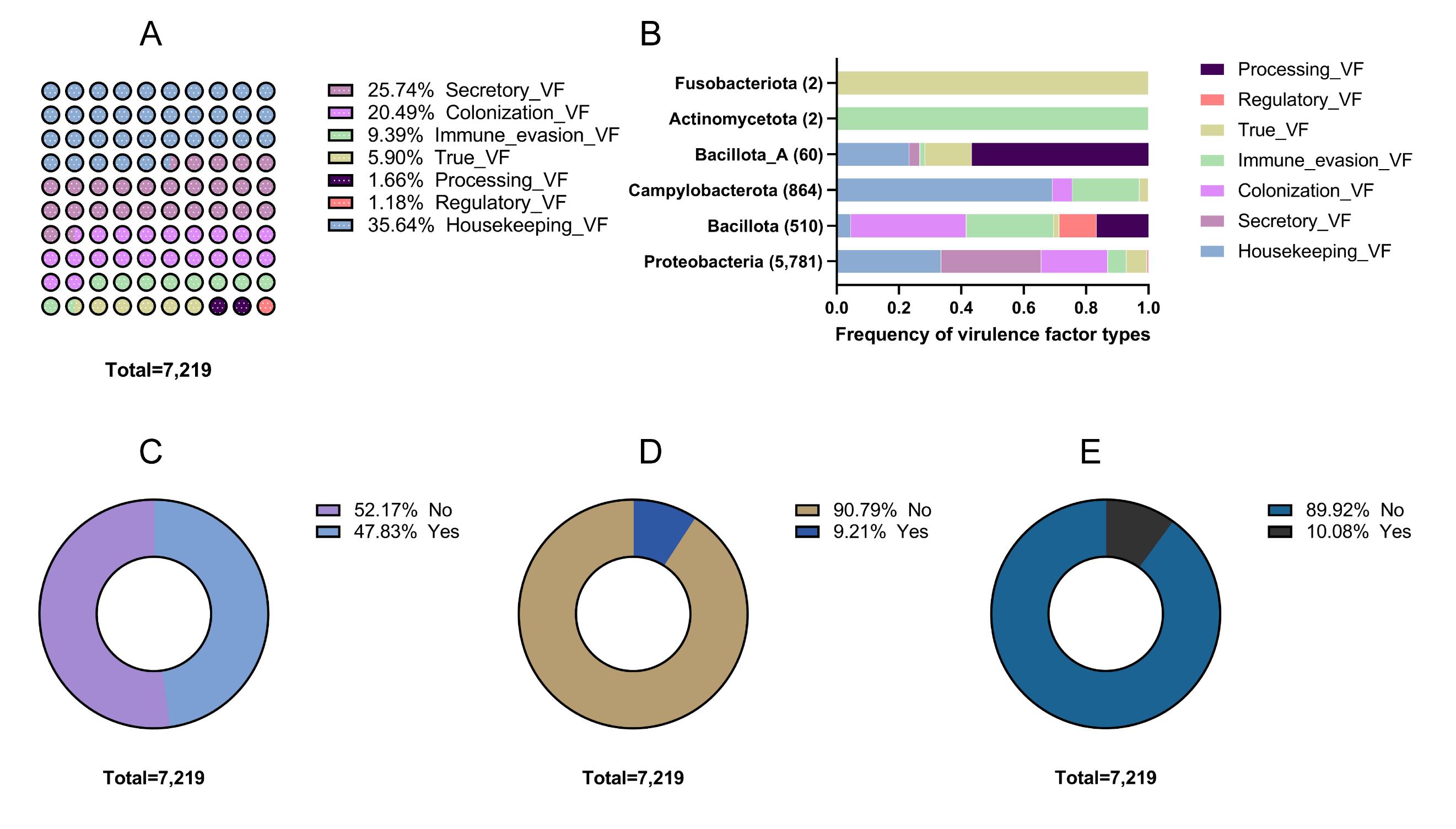


Supplementary Fig. 5. Migratory MAGs contain a diverse range of potential virulence factors.

A, The diversity of virulence factors identified, which are classified based on VFDB 2.0 classification. B, Frequency of virulence factors represented by each phylum. C, The distribution of species-specific virulence factors. D, The distribution of plasmid-borne virulence factors. E, The distribution of prophage-borne virulence factors.


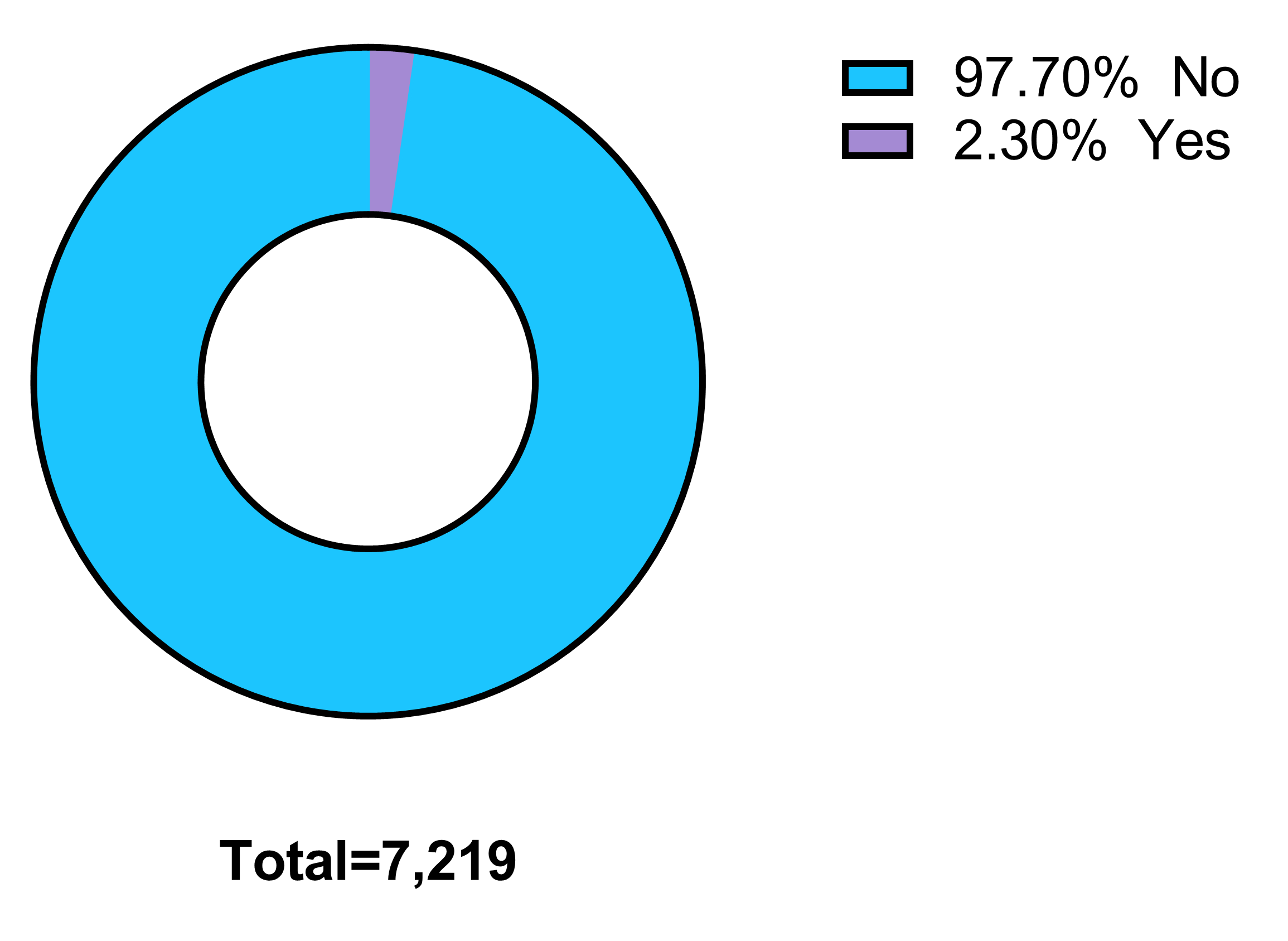


Supplementary Fig. 6. The distribution of virulence factors in unclassified species MAGs.


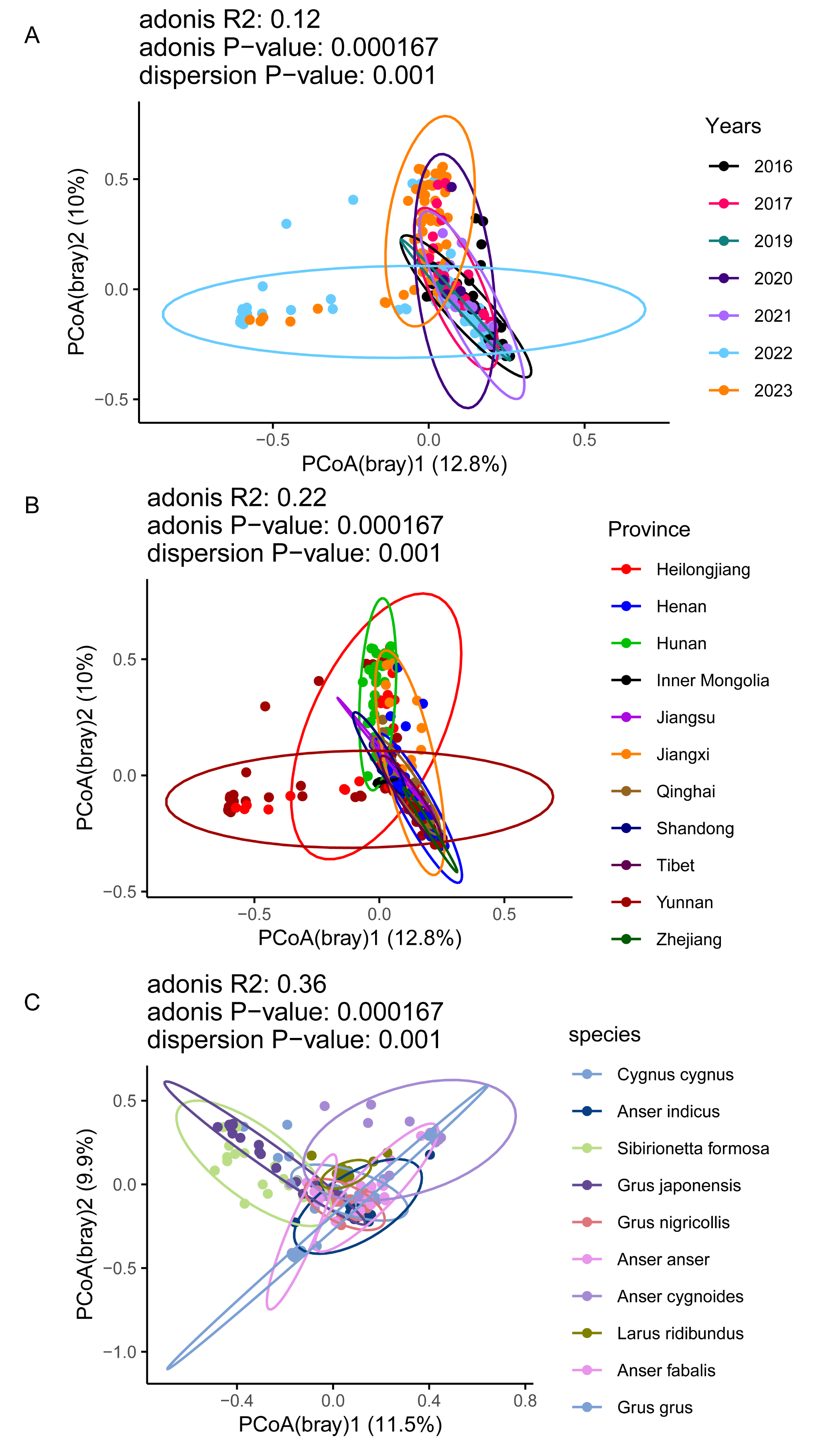
Supplementary Fig. 7. Principal coordinate analysis (PCoA) of bird microbiomes based on Bray–Curtis distance.

A, PCoA of bird gut bacterial communities from different sampling periods. B, PCoA of bird bacterial communities from different geographical locations. C, PCoA of bird bacterial communities between different species (the top 10).
